# A sophisticated case of division of labour in the trimorphic stamens of the *Cassia fistula* (Leguminosae) flower

**DOI:** 10.1093/aobpla/plab054

**Published:** 2021-08-27

**Authors:** Gabriella da Silva Saab, Vidal de Freitas Mansano, Anselmo Nogueira, Isabele Carvalho Maia, Pedro Joaquim Bergamo, Juliana Villela Paulino

**Affiliations:** 1 Departamento de Produtos Naturais e Alimentos, Faculdade de Farmácia, Centro de Ciências da Saúde, Universidade Federal do Rio de Janeiro (UFRJ), Rio de Janeiro, RJ 21941-902, Brazil; 2 Instituto de Pesquisas Jardim Botânico do Rio de Janeiro, DIPEQ, Rua Pacheco Leão 915, Rio de Janeiro, RJ 22460-030, Brazil; 3 Centro de Ciências Naturais e Humanas, Universidade Federal do ABC, São Bernardo do Campo, SP 09606-045, Brazil; 4 Programa de Pós-Graduação em Ecologia, Departamento de Biologia Vegetal, Instituto de Biologia, Universidade Estadual de Campinas (Unicamp), Barão Geraldo, Campinas, SP 13083-862, Brazil

**Keywords:** Buzz pollination, floral morphology, floral ontogeny, heteranthery, heteromorphic stamens, plant–pollinator interactions, pollen flower

## Abstract

Buzz-pollinated pollen flowers have pollen as the primary resource for pollinators and must deal with a conflict between the exploitation of pollen grains by bees and pollination success. It has been hypothesized that heterostemony allows division of labour between stamens as a solution to the pollen dilemma. To test the division of labour hypothesis, we chose *Cassia fistula*, which has a trimorphic androecium and analysed androecium development, pollen grain release mechanisms and visitor behaviour. We explored the reflectance of floral organs and carried out an exclusion experiment to test the attractiveness of each stamen morph to the bee species. Finally, we explored the structural, ultrastructural and functional variation between the pollen grains, including pollen viability across stamen morphs. The differences among the three stamen morphs, which is developed from two whorls of the stamen, are the first evidence of the division of labour in our study system. Large *Bombus* and *Xylocopa* bees actively and exclusively exploited the pollen grains from the central poricidal anthers generating pollen deposition on their bodies. The reflectance pattern of floral organs indicated a targeting of these large bees to the central anthers, corroborated by the anther manipulative experiment where only the exclusion of the anthers positioned in the flower centre, especially the intermediate stamens, reduced bee visits. Both results revealed a division of labour, in which the intermediate stamen morph was responsible for both floral attractiveness and pollen resources. Only the largest stamen morph produced germinable pollen grains, highlighting their role as pollinating stamens. The smallest stamen morph has a less clear function, likely representing an economy in pollen production for feeding function. Our findings suggest that the evolution of the trimorphic androecium is associated with division of labour in large pollen flowers and can represent a strong strategy for circumventing the pollen dilemma, optimizing the feeding function by reducing pollen grain investment from central anthers.

## Teaser

*Cassia fistula* flowers offer only pollen as food to the pollinator. Because of its three sets of stamens, we investigated the division of labour. As the same pollen cannot fertilize the plant and feed the pollinator, the idea of a set of stamens producing pollen for fertilization and another producing pollen to feed, is a refined strategy to mitigate this dilemma. The central stamens (intermediate and smallest stamens) are more attractive, producing inviable pollen grains, the bees’ choice for feeding. The largest stamens’ viable pollen grains are deposited on the bees’ back, confirming the division of labour in *C. fistula* flowers.

## Introduction

Pollen flowers are generally pollinated by bees and offer a surplus of pollen as an exclusive reward to pollinators instead of nectar and other floral resources (Vogel 1978). In pollen flowers, pollen grains (male gametophytes) act directly as the transport agent of the male gamete (Arroyo 1981; Pacini 2000; Lersten 2004; Barrett 2010), pollen grains also comprise the diet of larvae of insects, especially bees, as a primary floral resource sought out by pollinators (Agostini *et al.* 2014). In this context, pollinators may reduce the number of pollen grains available for fertilization since both plant reproduction and pollinator feeding functions are mutually exclusive (Vallejo-Marin *et al.* 2009; Barrett 2010; Agostini *et al.* 2014). Thus, in plants with pollen flowers increased pollinator attraction generates a trade-off with the amount of pollen destined as food for bees, a conflict between both functions of pollen as dispersal units and feeding pollen, a phenomenon known as the ‘pollen dilemma’. The plant must guard against excessive pollen collection by bees while it depends on these bees for pollination (Westerkamp 1996, 1997). Since in mutualism, each participant is trying to maximize the benefit received from its partner, there is a ‘tug of war’ between the interests of plants devoting more pollen to dispersal, and bees, in collecting more pollen as food (Papaj *et al.* 2017).

The recurrent and independent evolution of pollen flowers (*sensu*Vogel 1978) in multiple angiosperm families (Buchmann 1983) implies the appearance of strategies that circumvent the ‘pollen dilemma’ in these plant lineages (Westerkamp 1996, 1997). Indeed, plant species bearing pollen flowers have several features to deal with the ‘pollen dilemma’. For instance, pollen flowers can show polystemony (large number of stamens in relation to other floral parts) or larger anthers compared to flowers with other resources (Vogel 1978; Cruden 2000), both increasing pollen grains per flower to guarantee enough pollen for the two conflicting functions (Agostini *et al.* 2014). Additionally, pollen flowers commonly have poricidal anthers, controlling pollen release at each floral visit in a contingency strategy known as buzz pollination (Buchmann 1983; Harder and Barclay 1994; Vallejo-Marín 2019). Only specific groups of bees will be able to effectively vibrate flowers and reach the pollen (Pinheiro *et al.* 2014). Moreover, pollen flowers may exhibit a prominent heteromorphic androecium (heterostemony) as a mechanism for gradual pollen presentation (Kay *et al.* 2020) or associated with the division of labour of stamens (Luo *et al.* 2008, 2009; Vallejo-Marín *et al.* 2009, 2010; Paulino *et al.* 2013, 2016; Li *et al.* 2015; Mesquita-Neto *et al.* 2017) as the most refined mechanism to mitigate the ‘pollen dilemma’. In the last case, there is a clear difference in size, position and/or colour between a set of stamens that provides pollen as food for bees (feeding stamens) and other set producing pollen grains rarely exploited by bees, ensuring pollination and, consequently, egg cell fertilization (pollinating stamens) (Luo *et al.* 2009).

Although the morphological description and functional consequences of heterostemony have been reported for over 100 years (Müller 1881, 1882, 1883), experimental studies exploring the functioning of pollen flowers were only investigated much later (Macior 1968) and more recently in the context of the division of labour (e.g. Luo *et al.* 2009). Early evidence of the division of labour in the androecium are differences in stamen size and position associated with the bee behaviour within flowers (reviewed by Buchmann 1983). Some authors assumed that the division of labour should lead to the evolution of sterile pollen grains in the feeding anthers, a pattern corroborated in some plant systems (Paulino *et al.* 2016) but not in others (Nogueira *et al.* 2018; Velloso *et al.* 2018). More recently, studies of division of labour between stamen morphs incorporated details about the pattern of floral reflectance (Velloso *et al.* 2018; J.P. Basso-Alves et al., unpubl. data) and the pollen reserve content (Paulino *et al.* 2016; Velloso *et al.* 2018), increasing our understanding of the floral features related to the functioning of buzz pollination in pollen flowers. In sum, different kinds of evidence have been used to test the division of labour hypothesis. Still, few studies have attempted to integrate these approaches to hierarchically test the division of labour hypothesis in pollen flowers of the same plant species.

Most studies testing the division of labour hypothesis in pollen flowers have focused on representatives of Melastomataceae and Solanaceae that have two different stamen morphs (Luo *et al.* 2009; Vallejo-Marín *et al.* 2009, 2010; Papaj *et al.* 2017; Velloso *et al.* 2018). Thus, flowers with a trimorphic androecium are underexplored (Li *et al.* 2015), especially in other plant groups, such as Leguminosae, the third largest family of angiosperms (LPWG 2013, 2017; The Plant List 2013). In Leguminosae, ~30 genera of the 765 distributed in different legume clades have a heteromorphic androecium (Tucker 1996a; Rodriguez-Riaño *et al.* 1999; LPWG 2017). We chose the legume species *Cassia fistula* with large flowers and an androecium with three different stamen morphs to test the division of labour hypothesis among stamens. The large flowers of *C. fistula* have a marked spatial separation of stamens within the flower (Paulino *et al.* 2014) and a complex developmental pathway generating each of the three stamen morphs (see other *Cassia* species in Tucker 1996b). More specifically, we investigate four questions about floral ontogeny and androecium functionality: (i) Which developmental pathway results in forming a trimorphic androecium? (ii) Do bees visit the stamen morphs differently, indicating differential attractiveness across stamen morphs? (iii) Could the reflectance patterns and anther exclusion manipulation show the bees targeting the anthers of the flower centre hypothesized as feeding anthers? (iv) Do the feeding anthers produce less viable and starchless (lipid-rich) pollen grains than the pollination stamens? We do not have a clear expectation for the development pathway which gives rise to the three stamen morphs. We expect that the stamens from the flower centre are the most attractive to bees, bearing the most nutritious pollen grains for the feeding function, whereas abaxial stamens might provide viable pollen to guarantee the sexual plant reproduction.

## Materials and Methods

### Plant species and study area

**Figure 1.**
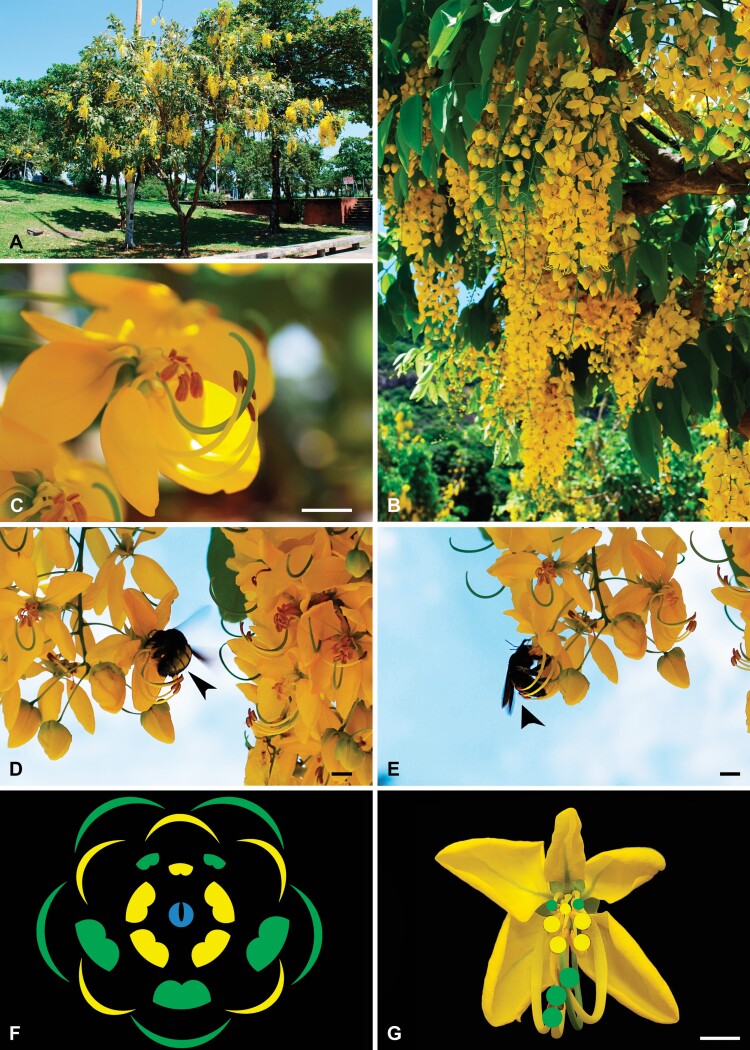
Images of a blossoming individual, inflorescences, flowers, and large bee species’ behaviour on flowers of *Cassia fistula*. (A) Blossoming individual in an urban area of Rio de Janeiro/RJ (Brazil). (B) Racemose pending inflorescences, subtended by abaxial bracts. (C) Complete, heterochlamydeous, pentamerous and zygomorphic flower in lateral view. (D and E) Behaviour of large bee species landing on the flower. Note the noto-sternotribic deposition of pollen grains due to the buzz pollination. The pollen grains of the largest stamens are deposited on the back of the bee (nototribic deposition, black arrows), while the bee vibrates the central stamens of the flower (intermediate and smallest stamens), collecting these pollen grains, which are deposited onto the ventral portion (sternotribic deposition). Note in the left image (D) that the stigma reaches the same region of the pollinator’s body in which the pollen from the largest stamens is deposited. (F and G) Floral diagram displaying the two stamen whorls, the antesepalous stamens opposite to the sepals and alternate to the petals (marked in green), and the antepetalous ones opposite to the petals and in alternate position to the antesepalous stamens (marked in yellow). Scale bars: C = 0.94 cm, D = 0.68 cm, E = 0.81 cm, G = 0.88 cm.

*Cassia fistula* is a species native to Asia from southern Pakistan east through India to Myanmar and south of Sri Lanka (Govindarajan 2009). It is a fast-growing tree species that reaches 5–6 m high, cultivated as an ornamental tree in tropical to subtropical climates, including several Brazilian regions. This plant is popularly known as ‘yellow shower tree’ due to its exuberant racemose pending inflorescences. Each of them is formed by several flowers and floral buds subtended by an abaxial bract (Fig. 1A–C). *Cassia fistula* flowers have no nectaries, and pollen grains enclosed within poricidal anthers are the only floral resource available for bee flower visitors. Floral anthesis lasts ~2 days.

The flowers of *C. fistula* are perfect, heterochlamydeous, pedicellate, pentamerous and zygomorphic (Fig. 1C, F and G). The calyx is dialysepalous, light green; the five sepals are hairy on the abaxial surface, orbicular, ~1.0 cm long. The corolla is light yellow, dialypetalous, composed of five petals ~2.5 cm long (Fig. 1C and G). The androecium is heteromorphic, formed by 10 free stamens arranged in three distinct morphs (Fig. 1C, F and G and Results section below). Here we chose to use the term ‘heterostemony’ rather than the commonly used ‘heteranthery’, because, in addition to differences in anther morphology, the three stamen morphs are characterized mainly by differences in position, size and shape of the filaments. The gynoecium is monocarpellate and rarely bicarpellate; the carpel is elongated and curved, green, stipitated, ~3.0 cm long. The ovary bears ~100 ovules; the curved style is cylindrical, ending in a subterminal and chambered stigma with marginal simple trichomes.

Our sampling effort included individuals and flowers of *C. fistula* from different localities in Brazil. Floral buds, opened flowers, anthers and pollen grains were collected and fixed from individuals cultivated at the Universidade Federal do Rio de Janeiro (Rio de Janeiro – RJ, Brazil) and Porangaba – SP, Brazil. To measure the size of each floral organ, we sampled 10 fresh flowers and immediately dissected their parts. Flowers for reflectance analyses were sampled from individuals at the Universidade Estadual de Campinas (Campinas – SP, Brazil). Observations of the visitation pattern and behaviour of bee species on flowers were evaluated in *C. fistula* individuals at the Universidade Federal do Rio de Janeiro (Rio de Janeiro – RJ, Brazil), and public areas of Santo André – SP, São Bernardo do Campo – SP and São Caetano do Sul – SP (Brazil). Similarly, the experimental tests of androecium function removing different stamen morphs were performed on the same individuals distributed on the public areas of Santo André (SP), São Bernardo do Campo (SP) and São Caetano do Sul (SP). Voucher specimens of *C. fistula* were deposited in the SPFR and RFA herbaria under the following numbers: J. V. Paulino *et al.* 13 (SPRF) and J. V. Paulino & G. S. Saab 15 and 16 (RFA), Nogueira A. 308 (HUFABC).

### Scanning electron microscopy analysis of the androecium at different development stages

Floral buds at different stages were collected and fixed in FAA 70 (formalin:acetic acid:alcohol; Johansen 1940), gradually dehydrated in an ethanol series and stored in 70 % ethanol. Then, the floral buds at several developmental stages were dissected to investigate the ontogeny of internal floral whorls, especially the androecium whorls, using a Leica MZ 75 stereomicroscope and a Leica S APO stereomicroscope. Finally, the samples were dried in a Bal-Tec CPD 030 critical point dryer (Bal-Tec AG, Liechtenstein). For scanning electron microscopy (SEM) analysis, the samples were mounted on aluminium stubs, placed on carbon tape and then coated with gold in an Emitech K550X sputter coater (Ashford, UK). Observations and illustrations were performed using Shimadzu SS-550 (FFCLRP/USP) and JEOL JSM-6490LV (CPBF-RJ) scanning electron microscopes at 15, 20 or 30 kV.

### Visitation pattern and behaviour of bee species on flowers

To describe the visitation pattern and behaviour of bee species, we systematically assigned 10 plants of *C. fistula* from RJ and SP and performed focal observations at 1-h intervals per inflorescence to track floral visits. The number of visits, the kind of bees and the floral parts touched by the bees were recorded between 07:00 and 17:00 h on different days during the flowering season, totalling 60 h of observations. We recorded the number of opened flowers per focal inflorescence and the stamen morph actively touched first by each bee visitor during pollen collection. We only performed the taxonomic identification of the two genera of largest bees able to touch the flower’s stigma due to their larger body sizes, considered potential pollinators. Other bee species were only recorded in our data set during our sampling. The terminology used for the buzz pollination studies and to describe the interaction between bees and flowers follows Vallejo-Marín (2019).

### Test of the visual attractiveness of different stamen morphs to bee visitors

The visual attractiveness of stamen morphs was initially explored by applying the bee vision model on the reflectance measurements of each floral organ. In this case, we evaluate whether the flower’s central stamens were considered the target of the bees’ visits according to the bees’ visual model. We measured the reflectance pattern for anthers and filaments of the largest and intermediate stamen morphs, not including the anthers and filaments of the smallest stamen morph due to their reduced size. The base and apex of the petal and the carpel were also measured for comparison. We measured 15 freshly opened flowers, each from a different *C. fistula* individual, using a USB4000 reflectance spectrophotometer (OceanOptics, Inc., Dunedin, FL, USA). Barium sulphate and a black chamber were used as white and black standards, respectively (Lunau *et al.* 2011). We took all reflectance measurements at a 45° angle using a 200–1100 nm UV/SR-VIS reflectance probe. We obtained colour variables in the bees’ subjective vision using the hexagon colour vision model (Chittka 1992). The spectral sensitivities of the bumblebee *Bombus terrestris* were used as bee model (Skorupski *et al.* 2007) since large bees are the most effective pollinators of plant species in the Cassinae tribe (Almeida *et al.* 2015). Standard daylight (D65) and green leaf (AV400) functions available in the pavo R-package were used in model calculations (Maia *et al.* 2013). Green leaf was used as background for petals, while upper petal reflectance was considered the background of stamens and carpels (see Fig. 1A and B). Each reflectance (filament, anther, upper and lower part of the petal and carpel) was modelled as a colour loci in the bee hexagon colour space following Chittka (1992). To this, we first calculated hyperbolic transformed quantum catches with von Kries correction using the vismodel function in pavo (Maia *et al.* 2013). Then, colour loci were plotted in the hexagon using the colspace function in pavo (Maia *et al.* 2013). The chromatic contrast with the relevant background (i.e. green leaves as background for petals, and petal colour for stamens and carpels) was calculated since it is a measurement related to the conspicuousness of the object and is essential for bee attraction at a close distance (Chittka 1992; Renoult *et al*. 2017; van der Kooi *et al.* 2019). It was calculated as the distance between each colour loci with the achromatic centre (Chittka 1992). We also calculated green contrasts as the specific contrast produced in the green photoreceptor minus a constant of 0.5 (Spaethe *et al.* 2001). The green contrast is an important parameter mediating long-distance attraction for bees (Giurfa *et al.* 1996). Then, to test if the colour contrasts differ among the floral parts (anther and filament of the largest and intermediate stamen morphs, the upper part of the petal, the lower part of the petal and carpel), the green contrast and the chromatic contrast with the relevant background were used as response variables in separate linear models. A Tukey *post hoc* contrast test was applied to investigate pairwise differences between the floral parts for each model (chromatic contrast and green contrast).

The visual attractiveness of stamen morphs was also exploited experimentally with the exclusion of different sets of stamens. *Cassia fistula* has large flowers with around 3 cm of diameter, and their stigma and anthers of the largest stamens are 2–3 cm far from the flower centre (Fig. 1A–C). According to the division of labour hypothesis applied to large-flowered species as *C. fistula*, we predicted that bees would be attracted to the central stamens (smallest and intermediate stamen morphs) hypothesized as ‘feeding stamens’. In contrast, we predicted these bees rarely exploited pollen grains directly from the largest stamen morphs hypothesized as ‘pollinating stamens’. This pattern would be expected, especially for large bee species, given the body of bee morphological fit to touch the stigma while vibrating the central anthers. Therefore, we carried out a manipulative experiment in eight individuals of *C. fistula* distributed in Santo André (SP), São Bernardo do Campo and São Caetano (SP) during the flowering period between December and February of 2017 and 2018. In these plants, we marked 59 inflorescences comprised of 414 flowers. Each inflorescence was covered with an organza bag to isolate pre-anthetic buds from any visitor before data collection. All inflorescences were divided between six treatments, and flowers of the same inflorescence received a unique treatment. We applied the following treatments to all flowers by inflorescence: 1—control flowers (without removal of any stamens); 2—removal of largest stamens; 3—removal of intermediate stamens; 4—removal of smallest stamens; 5—removal of intermediate and smallest stamens; 6—removal of all stamens of the flowers. The experiment was conducted on alternate days, choosing only sunny days. All treatments were applied in each sampling day to avoid any abiotic factor affecting a single treatment. After manipulating the stamens in each flower per inflorescence, we recorded all bee visits over time (census per hour), performing focal observations on flowers directly, without videotaping. We also recorded the behaviour of bee species on the flowers, including the contact between the bee and the stigma, the contact sites and the number of floral buzzes if they occurred. Thus, to test the treatment effect of stamen exclusion on the bee visitation pattern, we used generalized linear mixed models (GLMMs). We fitted two models with two different response variables: one with the total number of bee visits and the other only with the number of large bee visits (*Bombus* and *Xylocopa* visits) as response variables. Very few bees of the *Bombus* and *Xylocopa* genera remain in the urban environment, with *Xylocopa frontalis* and *Bombus morio* standing out almost exclusively in the largest cities of São Paulo (e.g. Agostini and Sazima 2003). In the absence of full species confirmation, we emphasized that these two bee species are traditionally seen visiting *C. fistula* flowers in the study area and were probably the only large bee species to visit the flowers during the experiment. Both response variables were treated as count data with a much higher variance than the average, and we used a negative binomial distribution in both models to avoid overdispersion (Hilbe 2011). Although the response variables are count data, they are still bee visit rates as they describe the number of visits over an hour per flower. In both models, the independent variable was the flower treatment varying the androecium structure (categorical factor with six levels). We also included inflorescences nested within each plant as a random variable in our models.

### Functionality and ultrastructure of pollen grains from different stamen morphs

As a preliminary indication of the cytoplasmic content of pollen grains, we carried out a staining test with the 1 % acetic carmine dye (Medina and Conagin 1964) applied on pollen grains from distinct stamen morphs. Pollen grains from all stamen morphs observed under a light microscope (Nova 606; Nova Optical Systems) stained red and were considered pollen grains with intact cytoplasmic content (Dafni and Firmage 2000). After this preliminary assessment, we performed an *in vitro* pollen tube growth test on pollen grains. We extracted pollen grains for at least five anthers from each stamen morph. We placed them in a culture medium containing distilled water, 12.5 % sucrose, 0.01 % boric acid, 1 mM CaCl_2_, 1 mM Ca(NO_3_)_2_, 1 mM MgSO_4_ and 0.5 % agar for at least 3 h in the dark at 25 °C (modified from Shivanna 2003). Subsequently, each sample of pollen grains was analysed under a light microscope. The image capture of pollen grains was obtained through a CMOS digital camera model BP 5.0, coupled to the Bioptika Microscope model B50. Germinated pollen grains with pollen tube length at least twice the pollen grain diameter were considered viable (Dafni and Firmage 2000). In each image, we counted the total number of pollen grains and those viable to assess the proportion of viable pollen grains. We modelled the proportion of viable pollen grains across stamen morphs using a GLMM with a binomial error distribution (see Crawley 2007 for details). Stamen morph was included in the modelling as a categorical fixed factor and flower identity as a random variable.

We also characterized the shape of pollen grains and hydration status at the presentation stage from different stamen morphs. For that, fresh pollen grains from at least five anthers of each stamen morph were sampled and immediately divided into two conditions: (i) immersion oil and (ii) water (Dafni *et al.* 2005; Pacini *et al.* 2006). If partially dehydrated (H_2_O less than 30 %), the shape of pollen grains would be originally ovoidal in the immersion oil and becoming spherical in the water (Pacini and Hesse 2004). On the other hand, if partially hydrated (H_2_O more than 30 %), the shape of pollen grains would be originally spherical in the immersion oil and water (Pacini and Hesse 2004). The samples of pollen grains from each stamen morph were mounted between slide and coverslip for observation in a Bioptika model B50 microscope coupled with a CMOS digital camera model BP 5.0 for image capture.

The ultrastructure of pollen grains from different stamen morphs was used to investigate the exine traits and pollen grains’ reserve content. For the SEM and transmission electron microscopy (TEM), we selected five anthers of each stamen morph from different pre-anthetic floral buds to perform the ultrastructure analysis of pollen grains. For SEM analysis, we applied the same procedures described in section (Scanning electron microscopy analysis of the androecium at different development stages). For TEM analysis, anthers were fixed in 5 % glutaraldehyde at 0.1 M, washed in Sörensen phosphate buffer (pH ¼ 7.2) and post-fixed under vacuum for ~1 h. Then, anthers were stored at 4 °C. Anthers were washed a second time in 0.1 M Sörensen phosphate buffer and fixed overnight in 1 % aqueous osmium tetroxide in phosphate buffer. We rewashed the anthers in phosphate buffer, gradually dehydrated them in acetone solution and embedded in Araldite 6005. Subsequently, the anthers were sectioned using a Leica Reichert ultramicrotome. Semi-thin sections (~1 µm) obtained using glass slides were stained with 0.05 % toluidine blue (O’Brien *et al.* 1964). After observing the materials and choosing the most representative ones, ultra-thin sections (~60 nm) were obtained using diamond blades and stained with 2 % uranyl acetate solution for 15 min (Watson 1958) and lead citrate for 15 min (Reynolds 1963). Observations and illustrations from different pollen grains for each stamen morph were made using a Hitachi H-7650 Transmission Electron Microscope (CENABIO–UFRJ) to characterize their reserve content. Images of the entire section of each pollen grain analysed were captured. The occurrence and proportion of amyloplasts and oleoplasts were used to describe the reserve content of the pollen grains from different stamen morphs, similar to floral nectary structure in some plant species (e.g. Guimarães *et al.* 2016). We quantified the percentage of amyloplasts in the entire ultra-fine section of each pollen grain. The occurrence of amyloplasts and oleoplasts (presence or absence) was modelled using a GLMM with a binomial error distribution. The stamen morph was included as a categorical fixed factor. The percentage of amyloplasts was modelled using a general linear model with a Gaussian error distribution, in which the stamen morph was included as a categorical fixed factor.

The ultrastructure of pollen grains was described following Halbritter *et al.* (2018). The term ‘pre-anthesis’ or ‘pre-anthetic’ is used here for floral buds that are in the stage immediately before flower opening (anthesis) and the term ‘post-anthesis’ for more advanced flowers, at a time when the bees have stopped visiting them.

### Statistical software and tools

All statistical analyses were performed in R 4.1 (Team R core 2009) with standard and additional packages, as follows: glmmADMB (Bolker *et al.* 2012), lme4 (Bates *et al.* 2016), nlme (Pinheiro *et al.* 2016), MASS (Ripley *et al.* 2015) and glmmTMB (Brooks *et al.* 2017). Pirateplots were generated using the package ‘yarrr’ version 0.1.5 (Phillips 2017).

## Results

### Floral ontogeny and morphology with an emphasis on the formation of the trimorphic androecium

**Figure 2.**
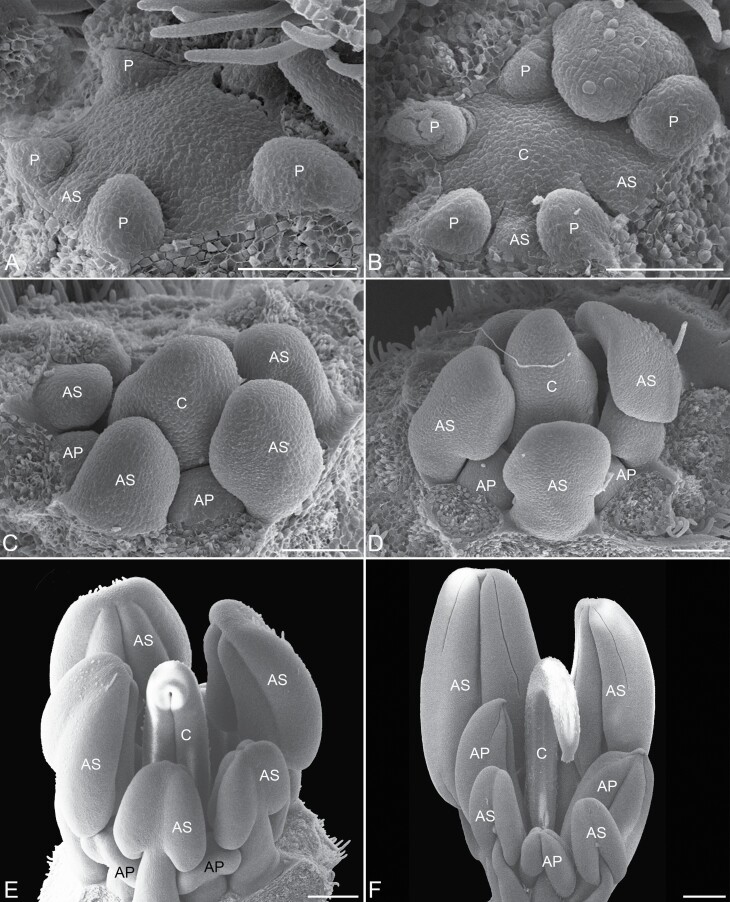
Androecium development in *Cassia fistula* (SEM). (A and B) Inception of the antesepalous stamen primordia, concomitant carpel initiation. (C) Inception of the antepetalous stamen primordia and the beginning of the antesepalous stamen elongation. (D) Stamen elongation and antesepalous anther differentiation. (E and F) Floral buds in a later stage, showing the elongation and differentiation of the two stamen whorls in the trimorphic androecium. Symbols: AP = antepetalous stamen; AS = antesepalous stamen; C = carpel; P = petal. Scale bars: A–D = 100 µm, E = 200 µm, F = 1 mm.

On the floral meristem, the androecium initiates in the antesepalous and antepetalous whorls (Fig. 2). The antesepalous stamens arise in a unidirectional order, starting with the median abaxial stamen, followed by the two lateral and finally, the adaxial ones (Fig. 2A–D). The antepetalous primordia of stamens are the last organs to appear on the floral meristem after carpel initiation, configuring the mixed acropetal floral organ formation (Fig. 2B). The filaments of the antesepalous stamens elongate before filaments of the antepetalous ones (Fig. 2C–E), concomitantly with the anther differentiation of all stamens. On the other hand, in the antepetalous whorl, the elongation of the filaments occurs when the anthers of the antesepalous stamens are in an advanced differentiation, especially the three abaxial ones (Fig. 2E and F). The filaments of the three abaxial antesepalous stamens extend longer than those of the two adaxial ones and form the largest set of stamens. In comparison, the two adaxial antesepalous stamens remain smaller and closer to the central region of the flower. In the antepetalous whorl, four stamens (two abaxial and two lateral) form the set of intermediate-sized stamens that surround the gynoecium base. The fifth adaxial antepetalous stamen remains small at the end of the floral development. Therefore, the three adaxial stamens of mixed origin include two lateral antesepalous and one median adaxial antepetalous and form the set of smallest stamens due to their reduced elongation (Fig. 1F and G).

**Figure 3.**
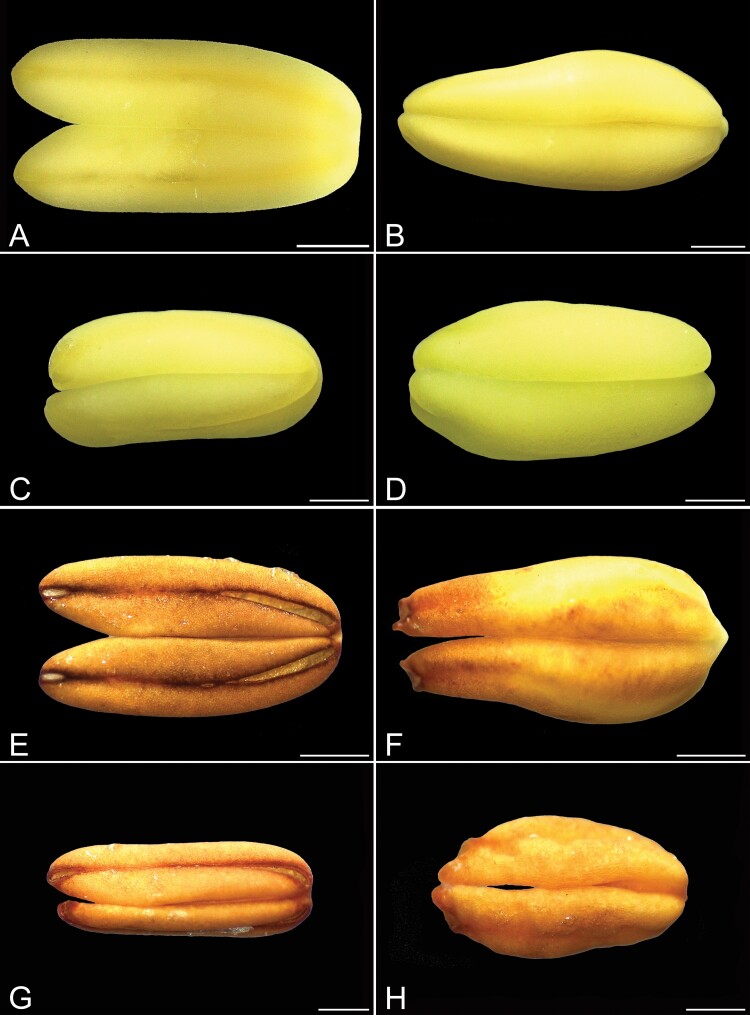
Dehiscence patterns of different types of anthers in flowers of *Cassia fistula.* (A) Anther of the largest stamens before anthesis. Note the longitudinal dehiscence line. (B) Anther of the intermediate stamens in pre-anthesis. (C) Anther of the lateral smallest stamens in pre-anthesis. Note the longitudinal dehiscence line. (D) Anther of the median smallest stamens in pre-anthesis. (E) Anther of the largest stamens at anthesis. Note the basal and apical opening, configuring the functionally poricidal dehiscence. (F) Poricidal dehiscence of the anther of the intermediate stamens at anthesis. (G) Anther of the lateral smallest stamens in anthesis. Note the basal and apical opening, configuring the functionally poricidal dehiscence. (H) Poricidal dehiscence of the anther of the median smallest stamens in anthesis. Scale bars: A and B, E and F = 1 mm, C and D, G and H = 0.5 mm.

At anthesis, flowers have the androecium with three stamen morphs characterized mainly by differences in position, size and shape of the filaments. Also, there are differences in the shape and size of the anthers (Fig. 1C and G; Table 1). Their features can easily distinguish the three morphs of stamens: (i) *the largest stamen morph*, consisting of three abaxial stamens, which accompanies the carpel in position, with greenish-yellow, long and sigmoidal filaments; (ii) *the intermediate stamen morph*, formed by four stamens that occupies a central position in the flower, around the base of the carpel, with slightly yellowish-green and sigmoidal filaments; and (iii) *the smallest stamen morph*, formed by three stamens that occupies the adaxial position, spatially close to the intermediate stamens, with greenish-yellow curved shape filaments. According to their origin, the anthers differ in shape, size, dehiscence and filament insertion (Table 1). Although all anthers have a longitudinal dehiscence line, all of them are functionally poricidal. The four anthers of the central intermediate stamen morph (Fig. 3B and F) and the middle stamen anther of the smallest stamen morph (antepetalous stamens) have basal poricidal dehiscence (Fig. 3D and H). Also, the anthers of the largest (Fig. 3A and E) and two lateral smallest stamen morphs (Fig. 3C and G) (antesepalous stamens) have small openings in the apical and basal portions of the dehiscence line and are considered functionally poricidal. Only the anthers of the intermediate morph have an apical appendage (Fig. 3B and F). Pollen grains release occurs when some bee species vibrate the stamens, characterizing the buzz pollination (see video available at figshare: doi.org/10.6084/m9.figshare.11786817.v1).

**Table 1.** Qualitative and quantitative comparison of androecium and pollen traits between the three distinct stamen morphs in flowers of *Cassia fistula*. Note that in the graphical representation of the flower (floral diagram), the sepals and the antesepalous stamens are represented in green, and the petals and the antepetalous stamens are represented in yellow.

	Largest stamen morphs 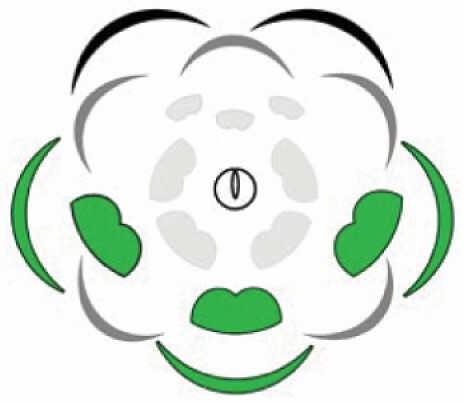	Intermediate stamen morphs 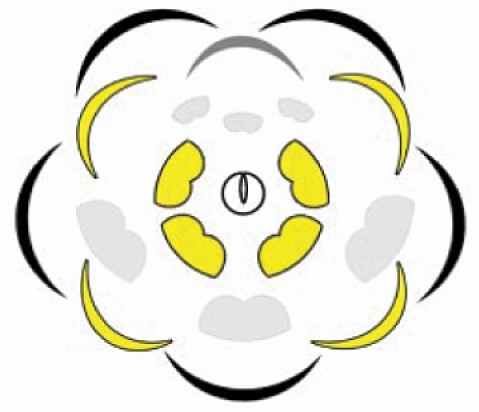	Smallest stamen morphs 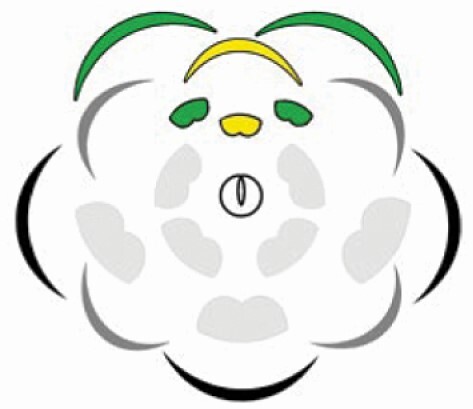
Stamen traits			
Origin	Antesepalous whorl	Antepetalous whorl	Mixed (two antesepalous + one antepetalous)
Anther form	Oblong to elliptical	Obovate	Obovate (median), oblong to elliptical (laterals)
Anther dehiscence	Functionally poricidal	Poricidal	Poricidal (median), functionally poricidal (laterals)
Filament insertion	Basifixed	Dorsifixed	Dorsifixed (median), basifixed (laterals)
Stamen length	4.4 cm	1.3 cm	0.7 cm (median) and 1.0 cm (laterals)
Filament length	4.0 cm	1.0 cm	0.4 cm (median) and 0.8 cm (laterals)
Anther length	0.4 cm	0.5 cm	0.3 cm (median) and 0.2 cm (laterals)
Pollen traits			
Number of pollen grains per anther	48 386.7 ± 12 308.3	52 628.6 ± 14 247.8	20 695.2 ± 6612.6
Number of pollen grains per morph	145 160.10	210 514.4	62 085.6
Equatorial axis length	31 μm ± 2.18	34 μm ± 1.42	24 μm ± 1.53
Polar axis length	30 μm ± 1.32	31 μm ± 1.73	22 μm ± 1.70

### The observational pattern of bee visitation in intact flowers

In general, all bee species visiting *C. fistula* flowers land on the intermediate and smallest stamens (central stamens) in 49 % of their visits but much more rarely touch the stigma during floral attendance. In contrast, large bees of the genera *Bombus* and *Xylocopa* responsible for 19.8 % of the total visits observed in *C. fistula* flowers (Fig. 1D and E) always touch the stigma when visiting flowers, acting as pollinators. In 100 % of visits, large bees land on the intermediate and smallest stamens and turn their back to the stigma and the largest stamens. The large bees bite the filaments of the central stamens and stop flying, just above the central anthers. While the large bees are trapped by the jaw in the filaments, their weight pulls the flower down. Almost concomitantly with this movement, the bee initiates vibrational behaviour. The contact of the stigma during landing on flowers can occur with the pollen of other flowers already deposited on the back of these large bees from previous visits (Fig. 1D and E). After landing on central stamens, the anthers of the largest stamens are positioned between the wings and the back of the abdomen of large bees. The large bees contract their thoracic muscles, performing the vibration, which results in the release of pollen grains from the central poricidal anthers close to their thorax (ventral region—sternotribic deposition). Simultaneously, these vibrations are transferred from different parts of the body of the bee to the specific parts of the flower. In particular, the closed of the wings and back abdomen of bees repeatedly touch the anthers of the largest stamens during vibrations, releasing the pollen grains from these anthers to the abdomen during floral attendance (nototribic deposition) (Fig. 1D and E). The pollen released from the largest stamens is deposited on the same region of the bee’s body reached by the stigma.

### The attractiveness of anthers indicates intermediate stamen morph as bee target in both observational and experimental evaluation

**Figure 4.**
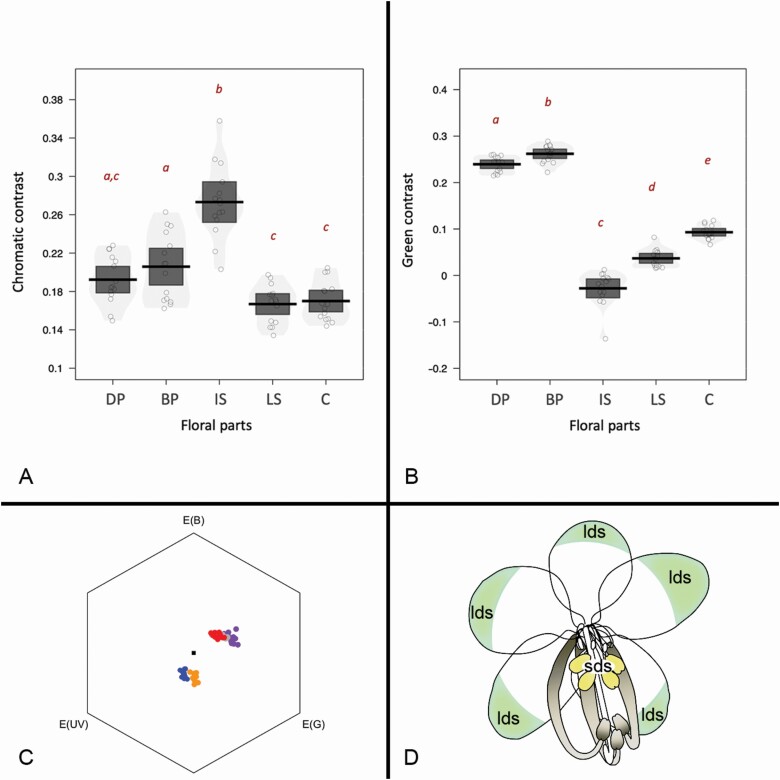
(A) Chromatic contrast against the relevant background of each floral part of *Cassia fistula*, estimated as distance between colour loci and achromatic centre (representing a leaf background) in the colour hexagon (following Chittka 1992). The chromatic contrast against the background is relevant at close distances, when a bee approaches and lands on a flower (Giurfa *et al.* 1996). (B) Green contrast of each floral part of *C. fistula*, estimated as the specific contrast produced in the bee green photoreceptor, minus a constant of 0.5 (following Spaethe *et al.* 2001). The green contrast is relevant at long distances, when bees are using only the visual information produced by the green photoreceptor (Giurfa *et al.* 1996). Distinct red letters indicate significant differences after Tukey *post hoc* tests (*P* < 0.05). (C) Bee hexagon visual model depicting the excitation (E) of ultraviolet (UV), green (G) and blue (B) photoreceptors. The black square represents the model’s achromatic centre, which corresponds to the background loci in the hexagon model. The blue points represent the modelled reflectance of the petals’ apex; orange points the petals’ base; red points the anthers of the largest stamen morph; grey the carpel; and purple indicates the anthers of the intermediate stamen morph. (D) Floral scheme representing how the floral reflectance directs the bee from the UV-reflecting apex of the petals to the UV-absorbing base of the petals (intrafloral UV pattern). These patterns direct the bee to the flower centre where the conspicuous intermediate stamens (feeding stamens, represented in yellow) are located, while the inconspicuous largest stamens (pollinating stamens, represented in grey) are ‘hide’ from the bee’s attention. Symbols: BP = basal part of the petal; C = carpel; DP = distal part of the petal; IS = intermediate stamen morph; lds = long-distance signal; LS = long stamen morph; sds = short-distance signal.

In the observational evaluation, we explored the reflectance pattern and colour vision model applied to floral organs. The distal part of the petals reflects more in ultraviolet (UV) wavelengths than the base of the petals, the latter being located close to the achromatic centre in the bee hexagon model (**see****Supporting Information—Fig. S1A****and****B**; Fig. 4C). The anthers of the largest and intermediate stamen morphs measured from *C. fistula* flowers generally reflect yellow wavelengths and absorb UV wavelengths. However, the anthers of the intermediate stamen morph reflect more in the yellow wavelength range than the anthers of the largest stamen morph **[see****Supporting Information—Fig. S1C****and****D****]**. Consequently, the anthers of the intermediate stamen morph show higher chromatic contrasts than the anthers of the largest stamen morph (*F*_4, 70_ = 33.96, *P* < 0.001). The *post hoc* contrasts revealed that colour conspicuousness (chromatic contrasts with the relevant background) was 0.07 to 0.11 higher for the intermediate stamens (0.27 ± 0.04), than for all other parts of the flower (Fig. 4A; **see****Supporting Information—Table S1**). The basal (0.21 ± 0.03) part of the petals showed the second-highest contrast with the relevant background, being different from all other parts of the flower except for the distal part (0.19 ± 0.02) of the petals. In the distal part of the petals, the largest stamens (0.17 ± 0.02) and carpel (0.17 ± 0.02) showed similar contrast with the relevant background values **[see****Supporting Information—Fig. S1E****and****F****]**. The green contrast also differed between floral parts (*F*_4, 70_ = 467.57, *P* < 0.001). All floral parts differed between them in green contrast (Fig. 4B; **see****Supporting Information—Table S2**). The basal part of the petals showed the highest green contrast (0.26 ± 0.02), followed by the distal part of the petals (0.24 ± 0.02). The androecium and carpel showed the lowest green contrast values. In order, carpels showed intermediate green contrast values (0.09 ± 0.01), followed by the largest stamens (0.04 ± 0.02) and intermediate stamens (−0.03 ± 0.04).

**Figure 5.**
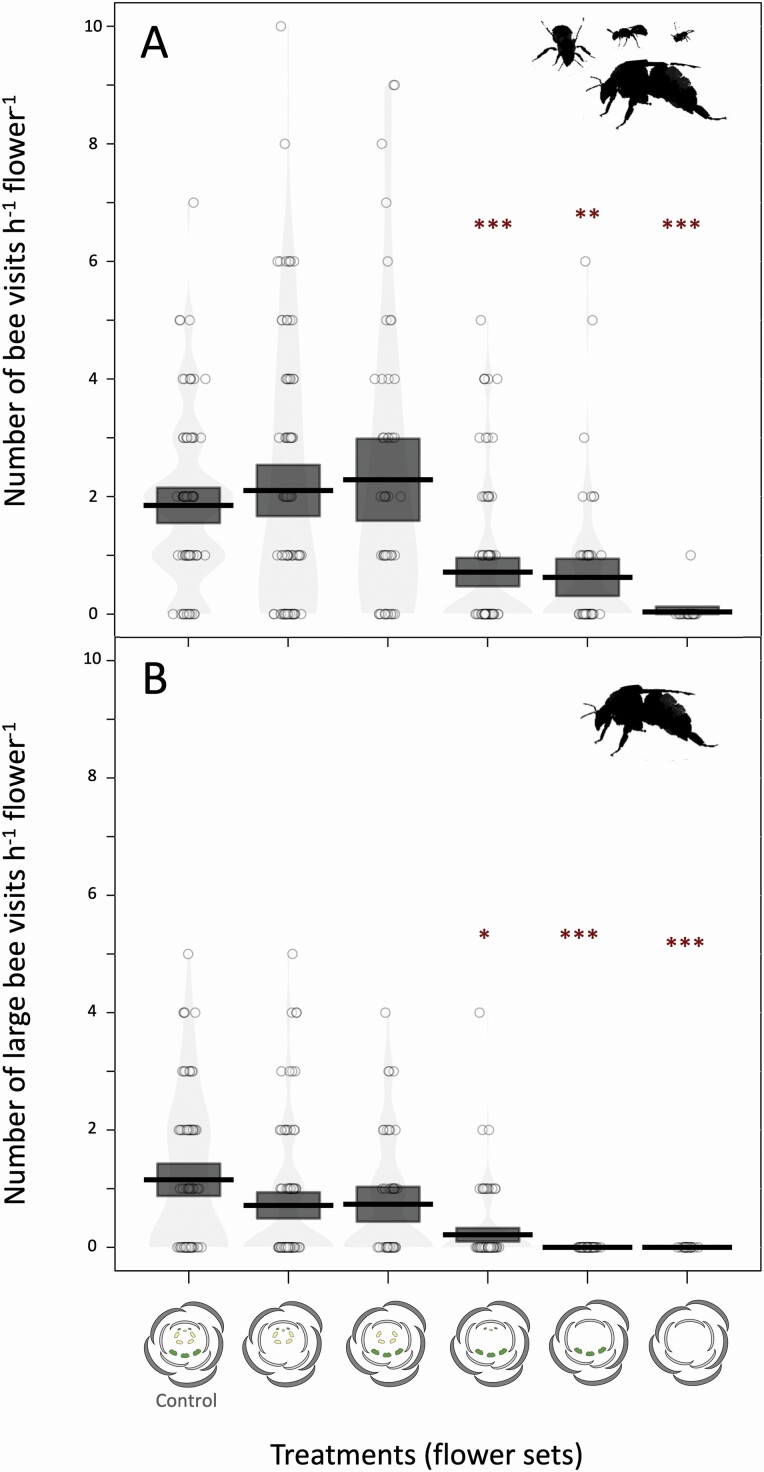
Bee visitation pattern on flowers of *Cassia fistula* under distinct anther removal treatments. (A) The average number of bee visits on flowers, including large and small bee species. (B) The average number of large bee visits on flowers, including only *Bombus* and *Xylocopa* species acting as pollinators during floral attendance. The asterisks highlight the treatments that had on average fewer bee visits when compared to the control flowers (*P = 0.013; **P < 0.01; ***P < 0.001). In general, the removal of the largest or smallest stamen morphs did not decrease the attractiveness of flowers when compared to intact flowers (control). In contrast, the removal of the intermediate stamen morph (feeding stamens) decreased the number of bee visits per hour (A), including the number of visits of large bee species (B). In each treatment, the black centre line indicates the mean, and the grey box encompasses the 95 % confidence interval. The unfilled jittered points represent the raw data, and the bean around the points is the smoothed density curve showing the full data distribution. Pirateplots were generated using the package ‘yarrr’ version 0.1.5 (Phillips 2017).

In the experimental evaluation, we excluded the different stamen morphs to test their effects on the rate of bee visits on each flower. In general, the bee visitation pattern differed among treatments applied to *C. fistula* flowers, with control unmanipulated flowers having on average two visits per flower per hour (Fig. 5A). The removal of the largest stamen morph (*z* = −0.84, *P* = 0.40) or the smallest stamen morph (*z* = −0.45, *P* = 0.65) did not decrease the number of bee visits when compared with intact flowers (control) (Fig. 5A). In contrast, removing the intermediate stamen morph decreased more than half of bee visits per hour (*z* = −3.33, *P* < 0.001; Fig. 5A). The visitation pattern of large bees of *Bombus* and *Xylocopa* similarly differed among treatments (Fig. 5B). For these large bees, control flowers have, on average, one visit per flower per hour, and the exclusion of intermediate stamen morph decreased the number of large bee visits compared with control flowers (*z* = −2.47, *P* = 0.013; Fig. 5B). In the extreme case of excluding anthers of both intermediate and the smallest stamen morphs, or the complete exclusion of anthers, flowers did not receive any visit of large bees.

### Comparison of nutritional and functional features of pollen grains produced in anthers of distinct stamen morphs

The anthers of the intermediate stamen morph have the highest production of pollen grains, while the smallest morph has 60 % fewer pollen grains per anther (Table 1). This pattern remains when comparing the number of pollen grains produced per stamen morph, i.e. the intermediate morph has the highest number of pollen grains, and the smallest morph has 70 % fewer pollen grains per flower (Table 1).

**Figure 6.**
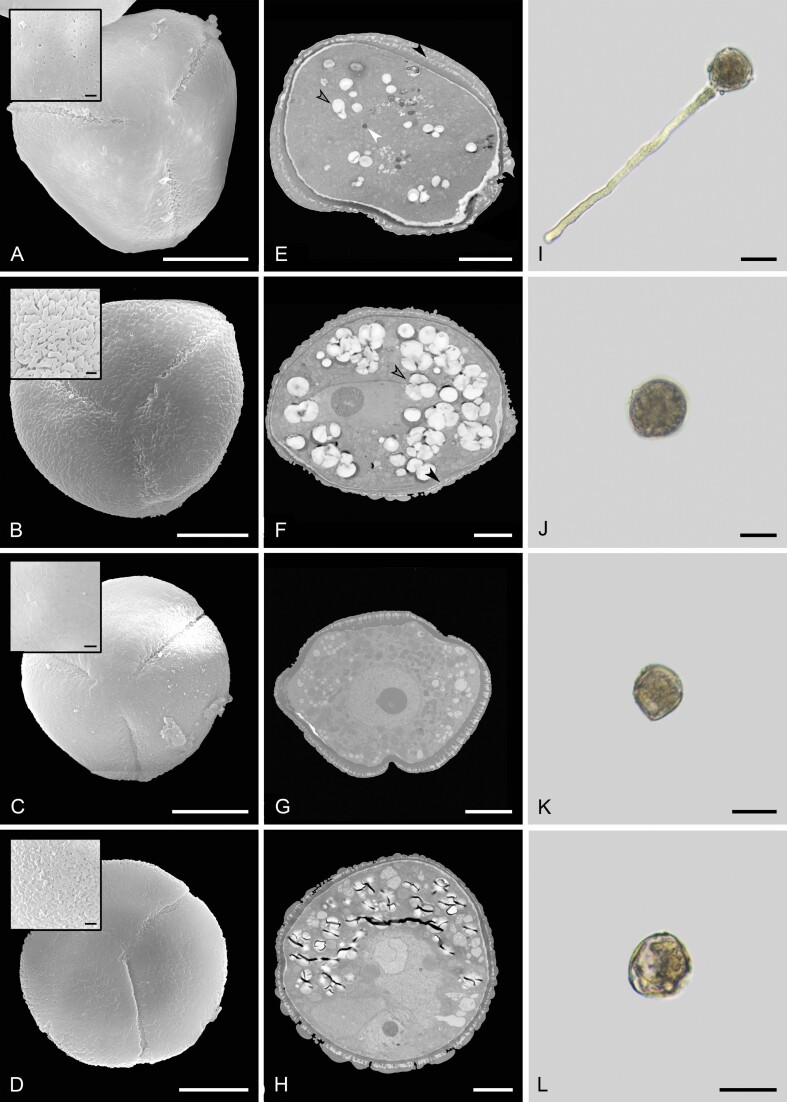
Characteristics of pollen grains from distinct stamen morphs in *Cassia fistula*. (A–D) Exine ornamentation patterns of pollen grains. (A and C) Pollen grains from largest and laterals stamens, respectively (antesepalous stamens), showing the perforate ornamentation. (B and D) Pollen grains from intermediate and median smallest stamens, respectively (antepetalous stamens), showing regulate ornamentation. (E–H) Cytoplasmic content of pollen grains. (E) Pollen grain from the largest stamens showing amyloplasts and oleoplasts. (F) Pollen grain from the intermediate stamens showing amyloplasts. (G) Pollen grain from the lateral smallest stamens showing few amyloplasts and oleoplasts and many organelles. (H) Pollen grain from the median smallest stamens, showing amyloplasts and several organelles. Black arrow outline = amyloplasts; white arrow = oleoplasts, black arrow = pollenkitt. (I–L) Pollen tube emission and growth under *in vitro* conditions. (I) Pollen grains of the largest stamen morph germinated and emitted pollen tubes under *in vitro* conditions. (J) Pollen grains of the intermediate stamen morph. (K) Pollen grains of the lateral smallest stamens. (L) Pollen grains of the median smallest stamens. Scale bars: A–D = 10 µm, Detailed images = 1 µm, E–H = 5 µm, I–K = 23 µm, L = 98 µm.

Pollen grains from all stamen morphs exposed to the immersion oil and water maintained the same spherical form in all stamen morphs. They were characterized as tricolporate pollen grains, with spheroidal form, presenting pollenkitt as the pollen coat, and been partially hydrated at the presentation. In the preliminary stained test, the three stamen morphs have pollen grains with cytoplasmic content. However, only pollen grains from the largest stamen morph are viable, exhibiting pollen tube emission and growth in the *in vitro* germination test (Fig. 6I–L). On average, anthers of the largest stamens have 48 % viable pollen versus a complete absence of viable pollen in anthers of the intermediate and smallest stamen morphs **[see****Supporting Information—Fig. S2****]**.

In SEM analysis, the largest axis of pollen grains from the largest and intermediate stamen morphs is quite similar, around 35 μm. In comparison, the pollen from the smallest stamen morphs is ~30 % smaller (~25 μm; Fig. 6A–D) (see also the **Supporting Information—Figs S3****and****S4**). Exine ornamentation of pollen grains differed among stamen morphs following the whorl origin. Pollen grains from anthers of the antesepalous whorl have perforated ornamentation (Fig. 6A and C), whereas pollen grains from anthers of the antepetalous whorl have rugulate ornamentation (Fig. 6B and D).

Similarly, pollen grains from distinct whorls show remarkable ultrastructural differences in TEM analysis. The pollen grains of the three largest and two smallest lateral stamens (antesepalous stamens) have vegetative cells containing both amyloplasts and oleoplasts as reserve content (Fig. 6E and G). In contrast, pollen grains of the four intermediate stamens and one median smallest stamen (antepetalous stamens) have vegetative cells containing a massive amount of amyloplasts (Fig. 6F and H) (see also the **Supporting Information—Fig. S5**). It is worth mentioning that in the pollen grains of the intermediate stamens, the amyloplasts stand out for filling ~50 % of the entire cytoplasmic content of the ultra-fine pollen section, forming large starch grains (Fig. 6F). On average, anthers of these intermediate stamens have pollen grains with amyloplast reserve five times greater than pollen grains of the largest stamens **[see****Supporting Information—Fig. S6****]**. The vegetative cells of pollen grains of the smallest stamen morph have the smallest amount of reserve content, showing the highest number of organelles and membrane systems (Fig. 6G and H). Finally, pollenkitt was observed in the tectum, and intercolumellar spaces of pollen grains of all stamen morphs, especially in pollen grains from the largest and intermediate stamen morphs (Fig. 6E–H).

## Discussion

Our results have shown that the development of the trimorphic androecium in flowers of the *C. fistula* is a consistent strategy leading to the division of labour among stamens, circumventing the pollen dilemma. The differences in size and position of the floral organs, especially the filaments between the three distinct morphs of stamens and the behaviour of large bee species on the flowers, are the first points that provide evidence of the division of labour among stamens. Large bees of *Bombus* and *Xylocopa* species have exclusively exploited the pollen grains of the central anthers in flowers of *C. fistula*, concomitantly touching the stigma during floral attendance. Larger bees are commonly the effective pollinators of large pollen flowers (e.g. Solís-Montero and Vallejo-Marín 2017), such as *C. fistula*, and large nectar flowers (e.g. Quinalha *et al.* 2017). The attractiveness of anthers based on our observational and experimental evaluations also indicates the intermediate stamen morph in the central region of the flower is the target of bee visitors and pollen feeding, corroborating our hypothesis that the central stamens compose a set of feeding stamens. Together, the combination of different evidence sources corroborates the division of labour hypothesis with the largest and intermediate stamen morphs serving pollination and feeding functions, respectively. Intriguingly, the smallest stamen morph has a less clear function in this flower. The smallest stamens of *C. fistula* flowers have small anthers with a lower number of pollen grains of a smaller size. This pattern indicates an incomplete development of these structures compared to the other stamen morphs. It probably represents a plant economy in pollen production as resources for pollinators among the central anthers. The pattern of pollen ultrastructure and functionality between stamen morphs also corroborated this interpretation. The largest and intermediate stamen morphs have pollen grains with abundant reserve content with amyloplasts and oleoplasts, and only amyloplasts, respectively. The tiny pollen grains from the smallest stamen morphs have little reserve content, i.e. with lower nutritional value, as if their development had been interrupted or delayed. Also, only pollen grains from the largest stamens can germinate and fertilize the ovules, highlighting their pollinating function, in contrast with the feeding function of the pollen grains from the intermediate stamen morph or pollen grains from the smallest stamen morph.

The functioning of *C. fistula* flowers is a more pronounced case of division of labour among stamens if compared to those observed in other species with trimorphic androecium in the subtribe Cassinae. For example, *Senna reniformis* showed fertile pollen grains produced by the anthers of all stamen morphs. Still, pollen grains from anthers of the largest and medium stamens had greater viability than those of the smallest ones (Mesquita-Neto *et al.* 2017). These results have important ecological implications for our understanding of pollen flowers’ evolution and buzz pollination, which are discussed below.

### Floral development, morphology and bee behaviour on a trimorphic androecium

In Leguminosae, the androecium is formed by two whorls, one with five antesepalous stamens and the other with five antepetalous stamens (Tucker 2003). The floral ontogeny of *C. fistula* showed a mixed developmental pathway in the formation of the trimorphic androecium. The largest and intermediate stamen morphs are composed of only one whorl each, antesepalous and antepetalous, respectively. The smallest stamen morph has a mixed origin formed by both antesepalous and antepetalous stamens. It is noteworthy that *C. fistula* deviates from the most common pattern of development found in other legume species with androecium heteromorphy. In such cases, the stamen heteromorphy is associated with the development of the two initial whorls separately, with the antesepalous whorl generating one stamen morph and the antepetalous whorl generating a second stamen morph (Paulino *et al.* 2016; De Barros *et al.* 2017). As a consequence of the mixed developmental pathway, androecium heteromorphy has previously been reported only in *Cytisus scoparius* with dimorphic androecium (Paulino *et al.* 2016) and now in *C. fistula* with trimorphic androecium.

At anthesis, the distance among anthers of the three stamen morphs is generated by the differential elongation of the filaments. This distance allows the bee species, especially the large bees, to be positioned between the largest stamen morph and the central stamen morphs of the flower. The morphological fit between the body of large bees and the anthers determines both the amount of pollen collected by the bee and the amount deposited on its body of then. The largest stamens with long filaments follow the curvature of the style so that the opening of the stigma and the anther touch the same region of the body of the floral visitor, as already observed in other species with heteromorphic stamens (Luo *et al.* 2009; Paulino *et al.* 2013, 2016; Mesquita-Neto *et al.* 2017). The pollen grains of these anthers are deposited on the dorsal region of the abdomen in large bees (nototribic deposition), considered a ‘safe site’, i.e. a place on the body from which the bees are not able to remove the pollen (Koch *et al.* 2017). Minnaar *et al.* (2019) suggest that the non-diffuse pollen placement on pollinator bodies can reduce the risk of pollen being groomed, avoiding its use to feed the bee’s larvae. The smallest and intermediate stamens have small filaments. Their anthers occupy a central position in the flower, easily accessible to the floral vibration behaviour of bees that collect these pollen grains (sternotribic deposition).

### Target colour pattern and differential attractiveness of stamen morphs

Among some types of yellow flowers, the presence of yellow carotenoids in the distal part of the petals is associated with UV reflectance, while the lower UV reflectance at the base of the petals is due to the presence of flavonoids that absorb UV (Thompson *et al.* 1972; Harborne and Smith 1978). These areas in the UV-absorbing petals, known as ‘nectar guides’, are invisible to humans but are visible to pollinating insects, functioning as a visual guide for them to the floral resources of the flower (Thompson *et al.* 1972). Several bee-pollinated yellow flowers have such an intrafloral UV pattern, also known as ‘bulls-eye’, enhancing bee attraction (Papiorek *et al.* 2016). In the pollen flowers of *C. fistula* (*sensu*Vogel 1978), such a UV pattern may act as a ‘pollen guide’, directing the pollinator to the central anthers. The greater reflectance of anthers in the intermediate stamen morph resulted in higher chromatic contrast than those of the largest ones or carpel. This higher reflectance indicates that intermediate stamens are visually more attractive to bees, contrasting with several plant species on which only the petals are visually more attractive (Teixeira *et al.* 2014). Importantly, we found that the largest stamens showed the lowest conspicuousness, which may ‘hide’ these anthers from the bee’s attention. This pattern is similar to the mechanism proposed to explain how flowers can avoid less effective bee visitors by displaying less attractive colours (Lunau *et al.* 2011; Bergamo *et al.* 2019; de Camargo *et al.* 2019; Chen *et al.* 2020).

The intense reflectance of the anthers from the central intermediate stamen morph, together with the intrafloral UV pattern produced by petals, reveals a kind of ‘target’ on these flowers (see Fig. 4D). It is already known that the intrafloral UV gradient within a flower directs the bees to the centre of the flowers (Rohde *et al.* 2013; Papiorek *et al.* 2016). The intermediate stamens’ central position with their high chromatic contrasts may drive bee attention since the contrast with the background is an important parameter determining bee attraction (Bergamo *et al.* 2016; Telles *et al.* 2017). Therefore, the whole floral colour pattern can perform the function of directing the bee to the anthers of the intermediate stamens (feeding stamens), responsible for supplying the pollinator’s feeding demand. The intrafloral UV gradient can explain the pollinator’s learned preference for the intermediate stamen morph in the experimental tests. Interestingly, we found this outer-to-inner pattern of contrast only when evaluating the chromatic contrast, indicating that only at close distances when a bee approaches the flower, the colour pattern directs the bees towards the central anthers (see van der Kooi *et al.* 2019 for a fruitful discussion about the context on which chromatic contrast is relevant). On the other hand, only the green contrast of petals was high, which indicates that petals should present a strong contrast to attract bees at long distances (Giurfa *et al.* 1996). Apart from intrafloral visual patterns, anther or pollen scent differences between stamen morphs may also direct bee foraging (Russell *et al.* 2017; Solís-Montero *et al.* 2018), contributing to the division of labour.

Our experiment of excluding different sets of stamens revealed a division of labour with only the intermediate stamen morph responsible for the floral attractiveness in *C. fistula* flowers. The exclusion of the intermediate stamen morph (feeding stamens) strongly decreased the number of bee visits on flowers, especially the large bee species behaving like pollinators. The visitation pattern of bees indicates that somehow most bees did not recognize flowers without the intermediate stamens as sources of pollen grains available for collection, in contrast with the findings in *Solanun houstonii* Dunal, in which the bees can flexibly increase pollen collection from pollinating anthers if pollen grains from short anthers (feeding anthers) make unavailable to the pollinator (Papaj *et al.* 2017). Floral traits, including anther size, are essential for bee attraction (Lunau *et al.* 2017) and probably played a crucial role in determining the bee visitation pattern in our experiments. This pattern corroborates the hypothesis of a division of labour between the largest and intermediate stamen morphs as pollination and feeding functions, respectively. However, the smallest stamen morph produces fewer and smaller pollen grains in *C. fistula* flowers. The reduced size of anthers in the smallest stamens may represent savings in pollen grain investment for attracting pollinators. This reduction would be an extreme case of division of labour after the functional segregation of pollinating and feeding anthers when the central stamens of the flower would be secondarily optimized for a less costly feeding function. Events of heterochrony would modify the developmental pattern of part of these stamens giving rise to the third stamen morph, the smallest in size, such as observed in flowers of *C. fistula* (present study) and *S. reniformis* (Mesquita-Neto *et al.* 2017). This pattern is quite different than observed in other legume species with two distinct stamen morphs (dimorphic androecium), such as *Swartzia dipetala* (Paulino *et al.* 2013), *C. scoparius*, *Lupinus* ‘The Governor’ (Paulino *et al.* 2016) and species of *Senna* (Luo *et al.* 2009), or species with a less clear distinction between stamen morphs as some *Chamaecrista* species (Nogueira *et al.* 2018).

### Functional and nutritional variation of pollen grains in a trimorphic androecium

Pollen viability tests showed that only pollen grains from the largest stamen morph germinate and form pollen tubes. Therefore, pollen grains from the intermediate and smallest stamen morphs are not functional for fertilization and could minimize production cost, decreasing the relative investments of these pollen grains related to feeding bee larvae (Vallejo-Marín *et al.* 2009, 2010). The division of labour in heterostemonous flowers could lead to the evolution of sterile pollen grains in the feeding anthers as an ultimate evolutionary step, such as previously reported in *Lupinus* (Paulino *et al.* 2016). In *Senna reniformis,* the viability of feeding pollen is reduced and could represent an intermediate stage in the evolution of sterile pollen (Mesquita-Neto *et al.* 2017).

Pollen grains of *C. fistula* were classified into two groups according to their vegetative cells cytoplasmic content in the ultrastructural analysis: starchy and starchless (Baker and Baker 1979). The variation of pollen grain nutritional quality is vital to the bee diet and could drive their preferences during floral attendance (Ruedenauer *et al.* 2020). Our data demonstrated that the pollen grains of the largest stamen morph and two lateral smallest stamens (originated from the antesepalous whorl) do not store high amount of starch and are not related to the stamens target by bees. Starchless pollen grains contain little or no starch, with large lipid and other carbohydrates due to the total or partial starch hydrolysis during the final phase of pollen maturation (Franchi *et al.* 1996; Pacini 2000; Pacini and Hesse 2004; Pacini *et al.* 2006). On the other hand, pollen grains of the intermediate stamens and smallest median one (originated from the antepetalous whorl) store starch and are called starchy pollen grains, with most starch not hydrolysed before pollen presentation (Franchi *et al.* 1996; Pacini 2000; Pacini and Hesse 2004; Pacini *et al.* 2006). So, the two types of pollen grain reserve are related to ontogenetic origin of the whorls.

Pollen represents a complex chemical mixture, and bee pollinators can differentiate nutritional quality between pollen types using non-volatile nutritional cues (e.g. Ruedenauer *et al.* 2015, 2020). Baker and Baker (1979) stated that flowers with pollen grains as the only reward offered to insects have lipid-rich small size pollen grains, suggesting bee preference to the lipid-rich pollen instead of starchy pollen. This expectation was not consistent with our results, given that the anthers of the intermediate stamens were more attractive to bees and had relatively large and abundant starchy pollen grains available for bees. This contradiction highlights the lack of experiments to evaluate the pollen-feeding animals’ preference for starchy or starchless pollen (Roulston and Cane 2000). Many bee species collect starchy pollen grains and digest the starch as a reserve (Roulston and Cane 2000). Besides, other legume species with heteromorphic androecium have predominantly starchy pollen-feeding (Paulino *et al.* 2016). It is tricky to evaluate whether the nutritional quality of the two types of pollen grains in *C. fistula* (starchy and starchless), defining the bees’ preference for the feeding anthers of the intermediate stamen morph. Other nutrients, such as proteins and fatty acids, can affect the bumblebee’s nutritional choice (Roulston and Cane 2000; Ruedenauer *et al.* 2020). Thus, further studies on nutritional pollen content in *C. fistula* and bee behaviour experiments are required to solve this matter.

From the plant perspective, differences in pollen grain cytophysiological features from different anthers can be related to their longevity during dispersion (Pacini 2000). The absence of water regulation (harmomegathy; Pacini 1990) as a mechanism for long-term viability in partially hydrated pollen grains (Pacini *et al.* 2006) reinforces the importance of other mechanisms maintaining pollen grain viability. Starchless and lipid pollen grains found in the anthers of the largest stamen morph (pollination stamens) are generally associated with more longevity than starchy pollen grains found in the intermediate (feeding stamens) in *C. fistula* flowers (Baker and Baker 1979). This pattern is due to the high cytoplasmic hydrolysed carbohydrate content as sucrose, fructose and glucose, enabling the maintenance of pollen grain viability (Pacini 2006). In particular, sucrose makes starchless grains more tolerant to desiccation in the final stages of pollen grains (Pacini 2006). Therefore, pollen grains from pollinating anthers probably maintain viability for a longer time than pollen grains produced by feeding anthers, favouring the success of the fertilization process.

## Conclusions

In this research, we investigated a sophisticated case of division of labour in flowers with a trimorphic androecium, as opposed to other plant examples with a dimorphic androecium (e.g. Luo *et al.* 2009; Paulino *et al.* 2016). The structural and functional differences within the androecium with three different stamens associated with bee behaviour support the division of labour hypothesis in *C. fistula* and represent a pollen grain economy for feeding functions due to differentiation of central anthers in two different morphs. Taken together, our results allow us to state that the largest abaxial stamens are the pollinating stamens and the intermediate stamens, to some extent with the smallest stamens, are the feeding stamens in flowers of *C. fistula*, corroborating the hypothesis initially proposed by Müller (1881). Importantly, we uncovered some unnoticed floral features that mediate such a division of labour: colour and ultrastructural differences, with prominent differences in inconspicuousness and cytoplasmic reserve and viability of the pollen grains between stamens. In our plant model, we found that the showy intermediate stamens and the smallest stamens produce pollen to supply the feeding demands of pollinators. In contrast, the inconspicuous largest stamens have starchless pollen grains for pollination. Our study highlights the importance of combining different sources of evidence to test the division of labour hypothesis of the androecium, including pollinator observation, floral manipulation and detailed characterization of stamens and pollen grains to reveal the functional mechanisms by which flowers circumvent the ‘pollen dilemma’.

## Supporting Information

The following additional information is available in the online version of this article—

**Figure S1.** Spectral reflectance of the floral parts of *Cassia fistula*.

**Figure S2.** Proportion of germinated pollen grains from anthers of different stamen morphs of *Cassia fistula* flowers.

**Figure S3.** Equatorial axis length of pollen grains from anthers of different stamen morph of flowers of *Cassia fistula*.

**Figure S4.** Polar axis length of pollen grains from anthers of different stamen morph of flowers of *Cassia fistula*.

**Figure S5.** Occurrence of amyloplasts and oleoplasts in pollen grains from anthers of different stamen morphs in *Cassia fistula* flowers.

**Figure S6.** Percentage of the area occupied by amyloplasts in one section of each pollen grain from anthers of different stamen morphs in *Cassia fistula* flowers.

**Table S1.** Effect sizes of the differences in chromatic contrast against the relevant background between floral parts of *Cassia fistula*.

**Table S2.** Effect sizes of the differences in green contrast between floral parts of *Cassia fistula*.

plab054_suppl_Supplementary_Material

plab054_suppl_Supplementary_Dataset_S1

plab054_suppl_Supplementary_Dataset_S2

## Data Availability

The raw data are available as Source 1: Complete database and Source 2: Complete script.

## Notes

**Form & Function. Chief Editor:** Kate McCulloh

## References

[CIT0001] Agostini K, Lopes AV, Machado IC. 2014. Recursos e Atrativos. In: Rech AR, Agostini K, Oliveira PE, Machado IC, eds. Biologia da Polinização. Rio de Janeiro, Brazil: Editora Projeto Cultural, 129–150.

[CIT0002] Agostini K, Sazima M. 2003. Plantas ornamentais e seus recursos para abelhas no campus da Universidade Estadual de Campinas, Estado de São Paulo, Brasil. Bragantia 62: 335– 343.

[CIT0003] Almeida NM, Cotarelli VM, Souza DP, Novo RR, Siqueira-Filho JA, Oliveira PE, Castro CC. 2015. Enantiostylous types of Cassiinae species (Fabaceae-Caesalpinioideae). Plant Biology 17:740–745. 25363754 10.1111/plb.12283

[CIT0004] Arroyo MTK. 1981. Breeding systems and pollination biology in Leguminosae. In: Polhill RM, Raven PH, eds. Advances in legume systematics. Part 2. Kew: Royal Botanic Garden, 723–769.

[CIT0005] Baker HG, Baker I. 1979. Starch in angiosperm pollen grains and its evolutionary significance. American Journal of Botany 66:591–600.

[CIT0006] Barrett SC. 2010. Darwin’s legacy: the forms, function and sexual diversity of flowers. Philosophical Transactions of the Royal Society of London. Series B, Biological Sciences 365:351–368. 20047864 10.1098/rstb.2009.0212 PMC2838255

[CIT0007] Bates D, Maechler M, Bolker B, Walker S, Christensen RHB, Singmann H, Dai B, Scheipl F, Grothendieck G, Green P, Fox J, Bauer A, Krivitsky PN. 2016. lme4: linear mixed-effects models using ‘Eigen’ and S4. R package version 1, 1–11.

[CIT0008] Bergamo PJ, Rech AR, Brito VL, Sazima M. 2016. Flower colour and visitation rates of *Costus arabicus* support the ‘bee avoidance’ hypothesis for red reflecting hummingbird pollinated flowers. Functional Ecology 30:710–720.

[CIT0009] Bergamo PJ, Wolowski M, Telles FJ, Brito VLG, Varassin IG, Sazima M. 2019. Bracts and long-tube flowers of hummingbird-pollinated plants are conspicuous to hummingbirds but not to bees. Biological Journal of the Linnean Society 126:533–544.

[CIT0010] Bolker B, Skaug H, Magnusson A, Nielsen A. 2012. Getting started with the glmmADMB package. R package 1–12.

[CIT0011] Brooks ME, Kristensen K, van Benthem KJ, Magnusson A, Berg CW, Nielsen A, Skaug HJ, Maechler M, Bolker BM. 2017. glmmTMB balances speed and flexibility among packages for zero-inflated generalized linear mixed modeling. The R Journal 9:378–400.

[CIT0012] Buchmann SL. 1983. Buzz pollination in angiosperms. In: Jones CE, Little RJ, eds. Handbook of experimental pollination biology. New York, NY: Van Nostrand Reinhold, 73–113.

[CIT0013] Chen Z, Niu Y, Liu CQ, Sun H. 2020. Red flowers differ in shades between pollination systems and across continents. Annals of Botany 126:837–848. 32478385 10.1093/aob/mcaa103 PMC7539362

[CIT0014] Chittka L. 1992. The colour hexagon: a chromaticity diagram based on photoreceptor excitations as a generalized representation of colour opponency. Journal of Comparative Physiology A 170:533–543.

[CIT0015] Crawley MJ. 2007. The R book. Chichester, UK: John Wiley & Sons, 942p.

[CIT0016] Cruden RW. 2000. Pollen grains: why so many? Plant Systematic and Evolution 222:143–165.

[CIT0017] Dafni A, Firmage D. 2000. Pollen viability and longevity: practical, ecological and evolutionary implications. In: Dafni A, Hesse M, Pacini E, eds. Pollen and pollination. Vienna, Austria: Springer, 113–132.

[CIT0018] Dafni A, Pacini E, Nepi M. 2005. Pollen and stigma biology. In: Dafni A, Kevan PG, Husband B, eds. Practical pollination biology. Cambridge, Canada: Enviroquest, 83–146.

[CIT0019] De Barros TC, Pedersoli GD, Paulino JV, Teixeira SP. 2017. In the interface of caesalpinioids and mimosoids: comparative floral development elucidates shared characters in *Dimorphandra mollis* and *Pentaclethra macroloba* (Leguminosae). American Journal of Botany 104:218–232. 28202455 10.3732/ajb.1600308

[CIT0020] de Camargo MGG, Lunau K, Batalha MA, Brings S, de Brito VLG, Morellato LPC. 2019. How flower colour signals allure bees and hummingbirds: a community-level test of the bee avoidance hypothesis. The New Phytologist 222:1112–1122. 30444536 10.1111/nph.15594

[CIT0021] Franchi GG, Bellani LM, Nepi M, Pacini E. 1996. Types of carbohydrate reserves in pollen: localization, systematic distribution and ecophysiological significance. Flora 191:1–17.

[CIT0022] Giurfa M, Vorobyev M, Kevan P, Menzel R. 1996. Detection of coloured stimuli by honeybees: minimum visual angles and receptor specific contrasts. Journal of Comparative Physiology A 178:699–709.

[CIT0023] Govindarajan M. 2009. Bioefficacy of Cassia fistula Linn. (Leguminosae) leaf extract against chikungunya vector, Aedes aegypti (Diptera: Culicidae). European Review for Medical and Pharmacological Sciences 13:99–103. 19499844

[CIT0024] Guimarães E, Nogueira A, Machado SR. 2016. Floral néctar production and nectary structure of a bee-pollinated shrub from Neotropical savanna. Plant Biology 18:26–36. 26194742 10.1111/plb.12370

[CIT0025] Halbritter H, Ulrich S, Grímsson F, Weber M, Zetter R, Hesse M, Buchner R, Svojtka M, Frosch-Radivo A. 2018. Illustrated pollen terminology. Springer.

[CIT0026] Harborne JB, Smith DM. 1978. Anthochlors and other flavonoids as honey guides in the Compositae. Biochemical Systematics and Ecology 6:287–291.

[CIT0027] Harder LD, Barclay RMR. 1994. The functional significance of poricidal anthers and buzz pollination: controlled pollen removal from Dodecatheon. Functional Ecology 8:509–517.

[CIT0028] Hilbe JM. 2011. Negative binomial regression, 2nd edn. New York, NY: Cambridge University Press.

[CIT0029] Johansen DA. 1940. Plant microtechnique. New York, NY: McGraw-Hill Book Co. Inc.

[CIT0030] Kay KM, Jogesh T, Tataru D, Akiba S. 2020. Darwin’s vexing contrivance: a new hypothesis for why some flowers have two kinds of anther. Proceedings of the Royal Society B. Biological Sciences 287:20202593. 10.1098/rspb.2020.2593 PMC7779490 33352073

[CIT0031] Koch L, Lunau K, Wester P. 2017. To be on the safe site - ungroomed spots on the bee’s body and their importance for pollination. PLoS One 12:e0182522. 28877178 10.1371/journal.pone.0182522 PMC5587100

[CIT0032] Lersten NR. 2004. Flowering plant embryology. Ames, Australia: Blackwell.

[CIT0033] Li JK, Song YP, Xu H, Zhang YW, Zhu JY, Tang LL. 2015. High ratio of illegitimate visitation by small bees severely weakens the potential function of heteranthery. Journal of Plant Ecology 8:213–223.

[CIT0034] LPWG [Legume Phylogeny Working Group]. 2013. Towards a new classification system for legumes: progress report from the 6th International Legume Conference. South African Journal of Botany 89:1–7.

[CIT0035] LPWG [Legume Phylogeny Working Group]. 2017. A new subfamily classification of the Leguminosae based on a taxonomically comprehensive phylogeny. Taxon 66:44–77.

[CIT0036] Lunau K, Konzmann S, Winter L, Kamphausen V, Ren ZX. 2017. Pollen and stamen mimicry: the alpine flora as a case study. Arthropod-Plant Interactions 11:427–447.

[CIT0037] Lunau K, Papiorek S, Eltz T, Sazima M. 2011. Avoidance of achromatic colours by bees provides a private niche for hummingbirds. The Journal of Experimental Biology 214:1607–1612. 21490268 10.1242/jeb.052688

[CIT0038] Luo Z-L, Gu L, Zhang D-X. 2009. Intrafloral differentiation of stamens in heterantherous flowers. Journal Systematic and Evolution 47:43–56.

[CIT0039] Luo Z, Zhang D, Renner SS. 2008. Why two kinds of stamens in buzz-pollinated flowers? Experimental support for Darwin’s division-of-labour hypothesis. Functional Ecology 22:794–800.

[CIT0040] Macior LW. 1968. An experimental study of the floral ecology of *Dodecatheon meadia*. American Journal of Botany 51:96–108.

[CIT0041] Maia R, Eliason CM, Bitton PP, Doucet SM, Shawkey MD. 2013. Pavo: an R package for the analysis, visualization and organization of spectral data. Methods in Ecology and Evolution 4:906–913.

[CIT0042] Medina DM, Conagin CHTM. 1964. Técnica citológica. Campinas, Brazil: Instituto Agronômico.

[CIT0043] Mesquita-Neto JN, Costa BKP, Schlindwein C. 2017. Heteranthery as a solution to the demand for pollen as food and for pollination—legitimate flower visitors reject flowers without feeding anthers. Plant Biology 19:942–950. 28762598 10.1111/plb.12609

[CIT0044] Minnaar C, Anderson B, de Jager ML, Karron JD. 2019. Plant-pollinator interactions along the pathway to paternity. Annals of Botany 123:225–245. 30535041 10.1093/aob/mcy167 PMC6344347

[CIT0045] Müller H. 1881. Two kinds of stamens with different functions in the same flower. Nature 24:307–308.

[CIT0046] Müller H. 1882. Two kinds of stamens with different functions in the same flower. Nature 26:30–30.

[CIT0047] Müller F. 1883. Two kinds of stamens with different functions in the same flower. Nature 27:364–365.

[CIT0048] Nogueira A, Valadão-Mendes LB, El Ottra JH, Guimarães E, Cardoso-Gustavon P, Quinalha MM, Paulino JV, Rando JG. 2018. Relationship of floral morphology and development with the pattern of bee visitation in a species with pollen-flowers, *Chamaecrista desvauxii* (Fabaceae). Botanical Journal of the Linnean Society 187:137–156.

[CIT0049] O’Brien TP, Feder N, McCcully ME. 1964. Polychromatic staining of plant cell walls by toluidine blue O. Protoplasma 59: 368–373.

[CIT0050] Pacini E. 1990. Harmomegathic characters of Pteridophyta spores and Spermatophyta pollen. In: Hesse M, Ehrendorfer F, eds. Morphology, development, and systematic relevance of pollen and spores. Vienna, Austria: Springer-Verlag, 53–69.

[CIT0051] Pacini E. 2000. From anther and pollen ripening to pollen presentation. Plant Systematics and Evolution 222:19–43.

[CIT0052] Pacini E, Guarnieri M, Nepi M. 2006. Pollen carbohydrates and water content during development, presentation, and dispersal: a short review. Protoplasma 228:73–77. 16937057 10.1007/s00709-006-0169-z

[CIT0053] Pacini E, Hesse M. 2004. Cytophysiology of pollen presentation and dispersal. Flora-Morphology, Distribution, Functional Ecology of Plants 199:273–285.

[CIT0054] Papaj DR, Buchmann SL, Russell AL. 2017. Division of labor of anthers in heterantherous plants: flexibility of bee pollen collection behavior may serve to keep plants honest. Arthropod-Plant Interactions 11:307–315.

[CIT0055] Papiorek S, Junker RR, Alves-dos-Santos I, Melo GAR, Amaral-Neto LP, Sazima M, Wolowski M, Freitas L, Lunau K. 2016. Bees, birds and yellow flowers: pollinator dependent convergent evolution of UV patterns. Plant Biology 18:46–55. 25703147 10.1111/plb.12322

[CIT0056] Paulino JV, Mansano VF, Prenner G. 2016. Evidence for division of labor and division of function related to the pollen release in Papilionoideae (Leguminosae) with a heteromorphic androecium. International Journal of Plant Sciences 177:590–607.

[CIT0057] Paulino JV, Mansano VF. Teixeira SP. 2013. Elucidating the unusual floral features of *Swartzia dipetala* (Fabaceae). Botanical Journal of the Linnean Society 173:303–320.

[CIT0058] Paulino JV, Prenner G, Mansano VF, Teixeira SP. 2014. Comparative development of rare cases of a polycarpellate gynoecium in an otherwise monocarpellate family, Leguminosae. American Journal of Botany 101:572–586. 24699538 10.3732/ajb.1300355

[CIT0059] Phillips N. 2017. *Yarrr: a companion to the e-book “yarrr!: the pirate’s guide to r”. Computer software manual (R package version 0.1.5)*. https://CRAN.R-project.org/package=yarrr (April 05, 2021).

[CIT0060] Pinheiro J, Bates D, DebRoy S, Sarkar D, Team RC. 2016. nlme: linear and nonlinear mixed effect models. R package version 3, 1–126. Available at: https://cran.r-project.org/web/packages/nlme/index.html (April 05, 2021).

[CIT0061] Pinheiro M, Gaglianone MC, Nunes CEP, Sigrist MR, Santos IA. 2014. Polinização por abelhas. In: Rech AR, Agostini K, Oliveira PE, Machado IC, eds. Biologia da Polinização. Rio de Janeiro, Brazil: Editora Projeto Cultural, 205–233.

[CIT0062] Quinalha MM, Nogueira A, Ferreira G, Guimarães E. 2017. Effect of mutualistic and antagonistic bees on floral resources and pollination of a savana shrub. Flora 232:30–38.

[CIT0063] Renoult JP, Kelber A, Schaefer HM. 2017. Colour spaces in ecology and evolutionary biology. Biological Reviews of the Cambridge Philosophical Society 92:292–315. 26468059 10.1111/brv.12230

[CIT0064] Reynolds ES. 1963. The use of lead citrate at high pH as an electron opaque stain in electronmicroscopy. Journal of Cell Biology 17:208–213. 10.1083/jcb.17.1.208 PMC2106263 13986422

[CIT0065] Ripley B, Venables B, Bates DM, Hornik K, Gebhardt A, Firth D. 2015. MASS: support functions and datasets for Venables and Ripley’s MASS. R package version 7, 3–45. Available at: https://https://cran.r-project.org/web/packages/MASS (April 05, 2021).

[CIT0066] Rodriguez-Riano T, Ortega-Olivencia A, Devesa JA. 1999. Types of androecium in the Fabaceae of SW Europe. Annals of Botany 83:109–116.

[CIT0067] Rohde K, Papiorek S, Lunau K. 2013. Bumblebees (*Bombus terrestris*) and honeybees (*Apis mellifera*) prefer similar colours of higher spectral purity over trained colours. Journal of Comparative Physiology A 199:197–210. 10.1007/s00359-012-0783-5 23224278

[CIT0068] Roulston TH, Cane JH. 2000. Pollen nutritional content and digestibility for animals. In: Dafni A, Hesse M, Pacini E, eds. Pollen and pollination. Vienna, Austria: Springer, 187–209.

[CIT0069] Ruedenauer FA, Raubenheimer D, Kessner-Beierlein D, Grund-Mueller N, Noack L, Spaethe J, Leonhardt SD. 2020. Best be(e) on low fat: linking nutrient perception, regulation and fitness. Ecology Letters 23:545–554. 31943632 10.1111/ele.13454

[CIT0070] Ruedenauer FA, Spaethe J, Leonhardt SD. 2015. How to know which food is good for you: bumblebees use taste to discriminate between different concentrations of food differing in nutrient content. The Journal of Experimental Biology 218:2233–2240. 26202778 10.1242/jeb.118554

[CIT0071] Russell AL., Buchmann SL, Papaj DR. 2017. How a generalist bee achieves high efficiency of pollen collection on diverse floral resources. Behavioral Ecology 28:991–1003.

[CIT0072] Shivanna KR. 2003. In vitro pollen germination and pollen tube growth. In: Shivanna KR, ed. Pollen biology and biotechnology. Enfield, NH: Science Publishers, 69–69.

[CIT0073] Skorupski P, Döring TF, Chittka L. 2007. Photoreceptor spectral sensitivity in island and mainland populations of the bumblebee, *Bombus terrestris*. Journal of Comparative Physiology A 193:485–494. 10.1007/s00359-006-0206-6 17333207

[CIT0074] Solís-Montero L, Cáceres-García S, Alavez-Rosas D, García-Crisóstomo JF, Vega-Polanco M, Grajales-Conesa J, Cruz-López L. 2018. Pollinator preferences for floral volatiles emitted by dimorphic anthers of a buzz-pollinated herb. Journal of Chemical Ecology 44:1058–1067. 30191434 10.1007/s10886-018-1014-5

[CIT0076] Solís-Montero L, Vallejo-Marín M. 2017. Does the morphological fit between flowers and pollinators affect pollen deposition? An experimental test in a buzz-pollinated species with anther dimorphism. Ecology and Evolution 7:2706–2715. 28428861 10.1002/ece3.2897 PMC5395427

[CIT0077] Spaethe J, Tautz J, Chittka L. 2001. Visual constraints in foraging bumblebees: flower size and color affect search time and flight behavior. Proceedings of the National Academy of Sciences of the United States of America 98:3898–3903. 11259668 10.1073/pnas.071053098 PMC31150

[CIT0075] Team R Core. 2009. 2017. RStudio: integrated development for R. Boston. Available at: http://www.rstudio.com.

[CIT0078] Teixeira SP, Marinho CR, Paulino JV. 2014. A Flor: aspectos morfofuncionais. In: Rech AR, Agostini K, Oliveira PE, Machado IC, eds. Biologia da Polinização. Rio de Janeiro, Brazil: Editora Projeto Cultural, 45–69.

[CIT0079] Telles FJ, Corcobado G, Trillo A, Rodríguez-Gironés MA. 2017. Multimodal cues provide redundant information for bumblebees when the stimulus is visually salient, but facilitate red target detection in a naturalistic background. PLoS One 12:e0184760. 28898287 10.1371/journal.pone.0184760 PMC5595325

[CIT0080] The Plant List. 2013. Version 1.1. Published on the Internet. http://www.theplantlist.org/ (1 January).

[CIT0081] Thompson WR, Meinwald J, Aneshansley D, Eisner T. 1972. Flavonols: pigments responsible for ultraviolet absorption in nectar guide of flower. Science 177:528–530. 5050486 10.1126/science.177.4048.528

[CIT0082] Tucker SC. 1996a. Trends in evolution of floral ontogeny in *Cassia sensu stricto, Senna*, and *Chamaecrista* (Leguminosae: Caesalpinioideae: Cassieae: Cassiinae): a study in convergence. American Journal of Botany 83:687–711.

[CIT0083] Tucker SC. 1996b. Stamen structure and development in legumes, with emphasis in poricidal stamens of caesalpinoid tribe Cassieae. In D’Arcy William G, Richard CK, eds. The anther: form, function, and phylogeny. Cambridge, UK: Cambridge University Press, 236–254.

[CIT0084] Tucker SC. 2003. Floral development in legumes. Plant Physiology 131:911–926. 12644644 10.1104/pp.102.017459 PMC1540291

[CIT0085] Vallejo-Marín M. 2019. Buzz pollination: studying bee vibrations on flowers. The New Phytologist 224:1068–1074. 30585638 10.1111/nph.15666

[CIT0086] Vallejo-Marín M, Da Silva EM, Sargent RD, Barrett SC. 2010. Trait correlates and functional significance of heteranthery in flowering plants. The New Phytologist 188:418–425. 20819173 10.1111/j.1469-8137.2010.03430.x

[CIT0087] Vallejo-Marín M, Manson JS, Thomson JD, Barrett SC. 2009. Division of labour within flowers: heteranthery, a floral strategy to reconcile contrasting pollen fates. Journal of Evolutionary Biology 22:828–839. 19320798 10.1111/j.1420-9101.2009.01693.x

[CIT0088] van der Kooi CJ, Dyer AG, Kevan PG, Lunau K. 2019. Functional significance of the optical properties of flowers for visual signalling. Annals of Botany 123:263–276. 29982325 10.1093/aob/mcy119 PMC6344213

[CIT0089] Velloso MDSC, Brito VLGD, Caetano APS, Romero R. 2018. Anther specializations related to the division of labor in Microlicia cordata (Spreng.) Cham. (Melastomataceae). Acta Botanica Brasilica 32:349–358.

[CIT0090] Vogel S. 1978. Evolutionary shifts from reward to deception in pollen flowers. In: Richards AJ, ed. The pollination of flowers by insects. London: Academic Press, 89–96.

[CIT0091] Watson ML. 1958. Staining of tissue sections for electron microscopy with heavy metals. The Journal of Biophysical and Biochemical Cytology 4:475–478. 13563554 10.1083/jcb.4.4.475 PMC2224499

[CIT0092] Westerkamp C. 1996. Pollen in bee-flower relations some considerations on melittophily. Botanica Acta 109:325–332.

[CIT0093] Westerkamp C. 1997. Flowers and bees are competitors-not partners. Towards a new understanding of complexity in specialised bee flowers. In: VII International Symposium on Pollination 437, 71–74.

