# Supplementary material for: A sophisticated case of division of labour in the trimorphic stamens of the Cassia fistula (Leguminosae) flower
Source: AoB Plants. 2021 Aug 27;13(5):plab054. doi: 10.1093/aobpla/plab054 (PMC8420109; doi:10.1093/aobpla/plab054)
Supplement: plab054_suppl_Supplementary_Material [file plab054_suppl_supplementary_material.pdf]

Supplemental Material

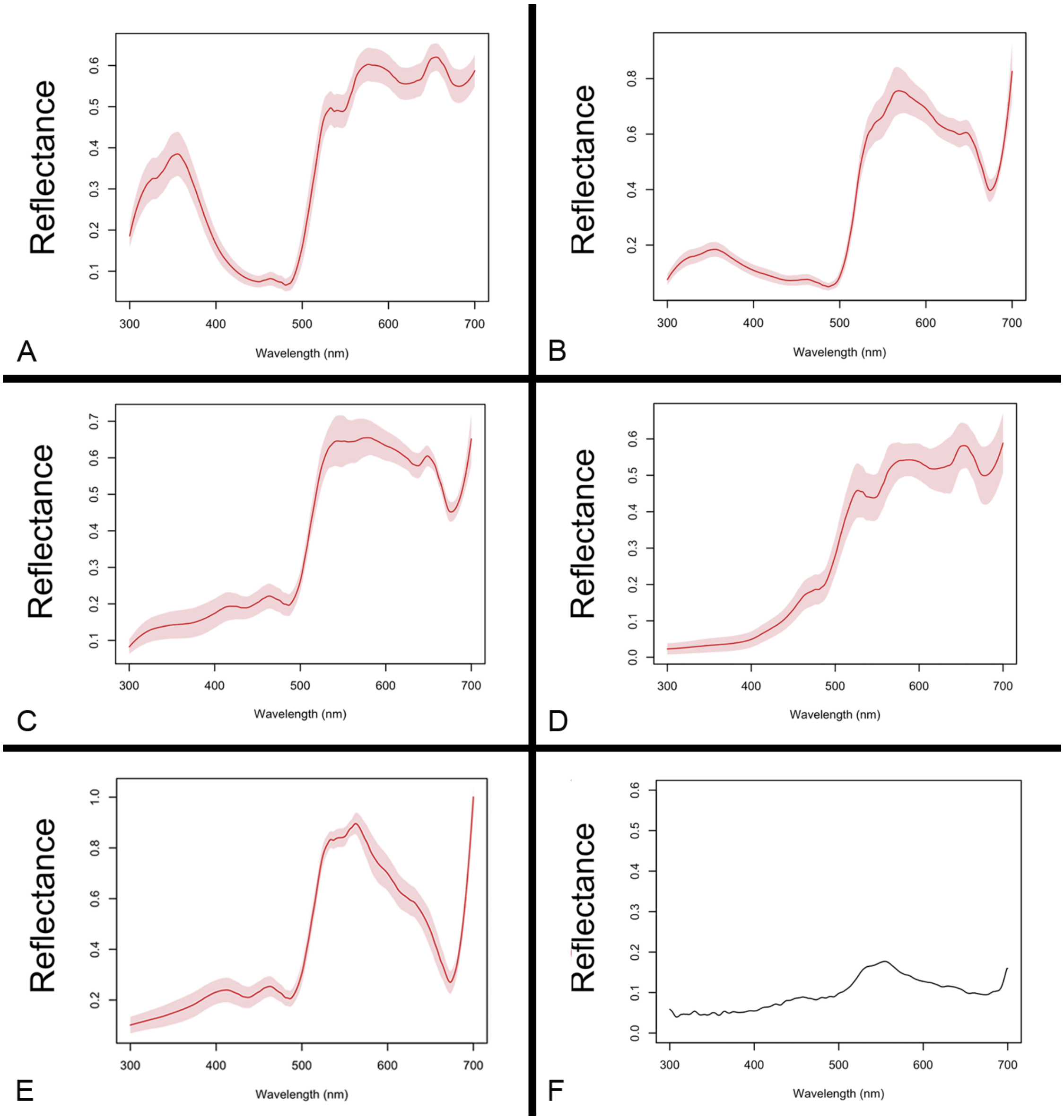

Figure S1: Spectral reflectance of the floral parts of *Cassia fistula* - A: Distal part of the petal. B: Basal part of the petal. C: Intermediate stamen morph. D. Largest stamen morph. E. Carpel. F. Leaf background reflectance (standard AV400). The leaf background reflects mostly in the green range, following the pattern reported for most plant species.

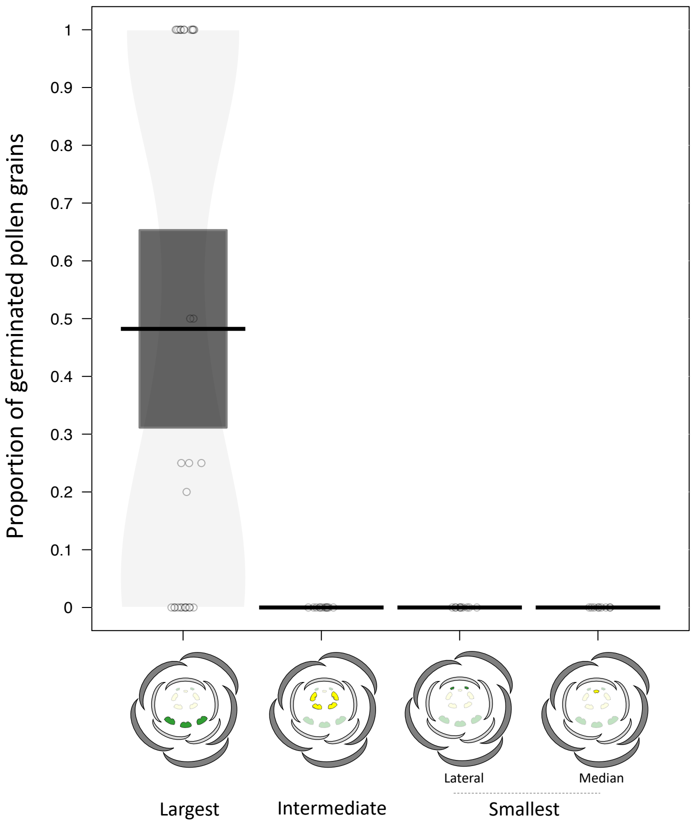

Figure S2: Proportion of germinated pollen grains from anthers of different stamen morph of flowers of *C. fistula*. The black centre line indicates the mean in each treatment, and the grey box encompasses the 95% confidence interval. The unfilled jittered points represent the raw data, and the bean around the points is the smoothed density curve showing the entire data distribution. Pirateplots were generated using the package 'yarrr' version 0.1.5 (Phillips 2017).

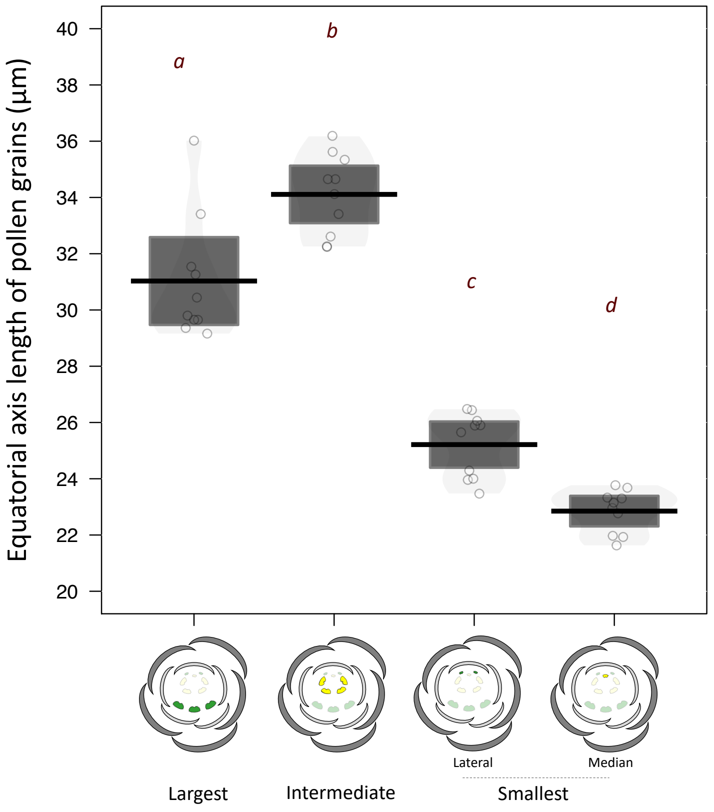

Figure S3: Equatorial axis length of pollen grains from anthers of different stamen morph of flowers of *C. fistula*. Different letters highlight differences in the equatorial axis length of pollen grains from anthers of distinct stamen morphs ($p < 0.001$). The black centre line indicates the mean in each treatment, and the grey box encompasses the 95% confidence interval. The unfilled jittered points represent the raw data of pollen grains, and the bean around the points is the smoothed density curve showing the entire data distribution. Pirateplots were generated using the package 'yarrr' version 0.1.5 (Phillips 2017).

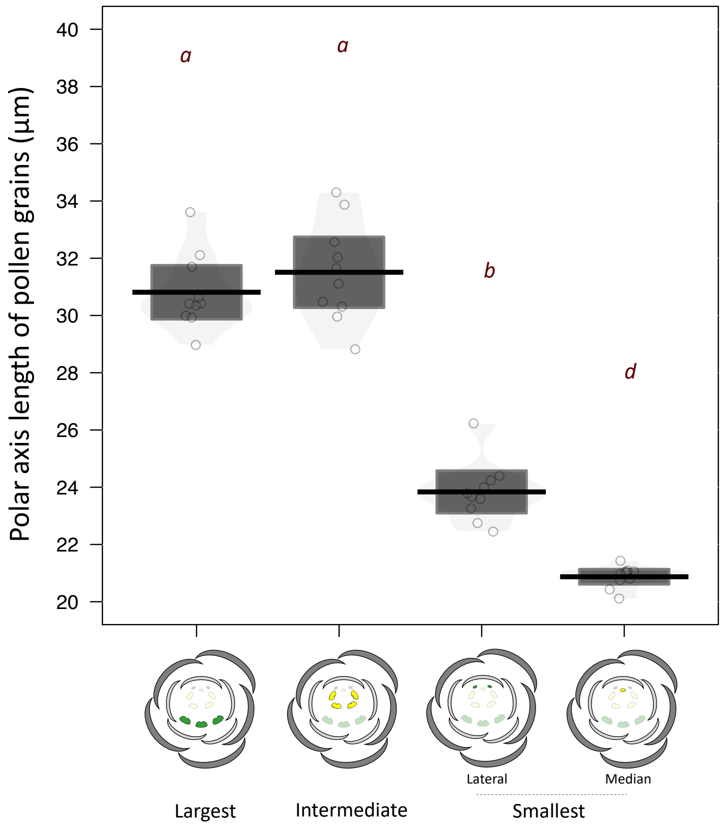

Figure S4: Polar axis length of pollen grains from anthers of different stamen morph of flowers of *C. fistula*. Different letters highlight differences in the polar axis length of pollen grains from anthers of distinct stamen morphs ($p < 0.001$). The black centre line indicates the mean in each treatment, and the grey box encompasses the 95% confidence interval. The unfilled jittered points represent the raw data of pollen grains, and the bean around the points is the smoothed density curve showing the entire data distribution. Pirateplots were generated using the package 'yarrr' version 0.1.5 (Phillips 2017).

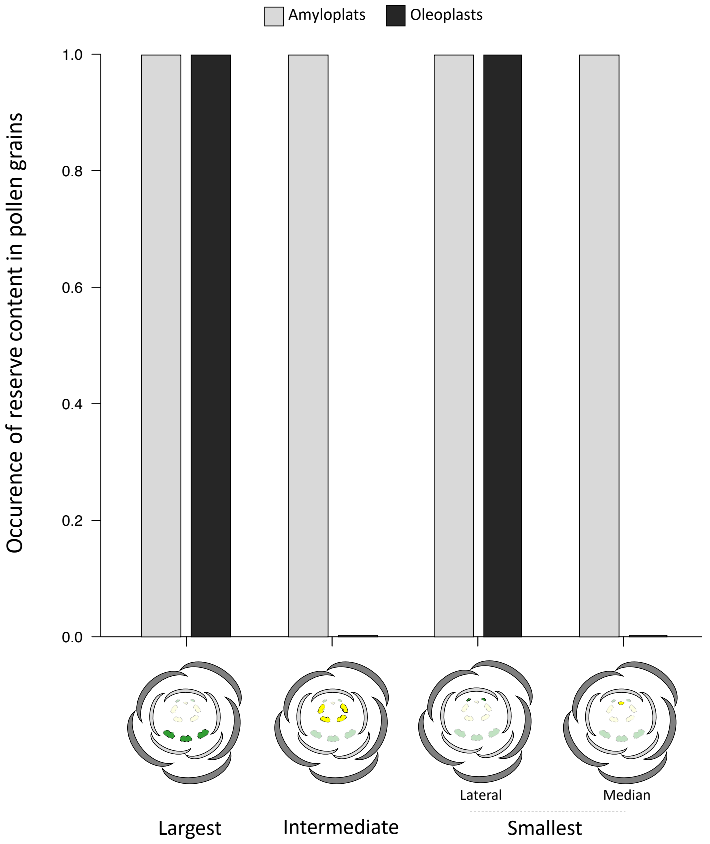

Figure S5: Occurrence of amyloplast (light grey) and oleoplasts (black) in pollen grains from anthers of different stamen morphs in *C. fistula* flowers. In our sampling, pollen grains from all anthers had amyloplasts. In contrast, oleoplasts were encountered only in pollen grains from the three largest and two smallest lateral stamen morphs (antesepalous stamens).

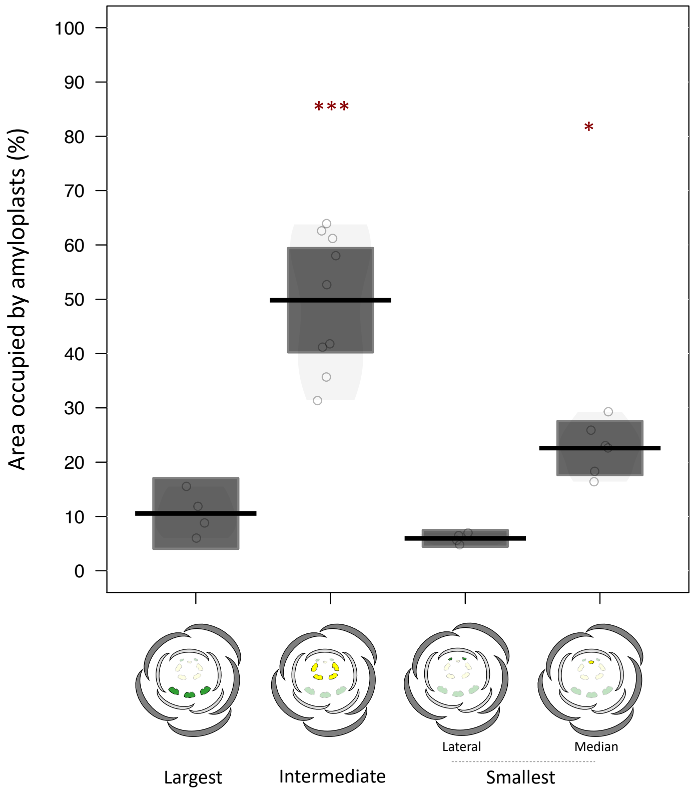

Figure S6: Percentage of the area occupied by amyloplasts in one section of each pollen grain from anthers of different stamen morph in *C. fistula* flowers. The asterisks highlight the stamen morphs with pollen grains that had, on average, a higher percentage of amyloplasts when compared to the largest stamen ("*" $p < 0.05$; "***" $p < 0.001$). The black centre line indicates the mean in each treatment, and the grey box encompasses the 95% confidence interval. The unfilled jittered points represent the raw data, and the bean around the points is the smoothed density curve showing the entire data distribution. Pirateplots were generated using the package 'yarrr' version 0.1.5 (Phillips 2017).

Table S1: Effect sizes of the differences in chromatic contrast against the relevant background between floral parts of *Cassia fistula*. Distinct red letters indicate significant differences after Tukey post-hoc tests ($p < 0.05$). dp = distal part of the petal; bp = basal part of the petal; is = intermediate stamen morph; ls = long stamen morph; c = carpel.

	**dp**	**bp**	**is**	**ls**	**c**
**dp**	-				
**bp**	0.01	-			
**is**	0.08	0.07	-		
**ls**	0.03	0.04	0.11	-	
**p**	0.02	0.04	0.11	0.01	-

Table S2: Effect sizes of the differences in green contrast between floral parts of *Cassia fistula*. Distinct red letters indicate significant differences after Tukey post-hoc tests ($p < 0.05$). dp = distal part of the petal; bp = basal part of the petal; is = intermediate stamen morph; ls = long stamen morph; c = carpel.

	**dp**	**bp**	**is**	**ls**	**c**
**dp**	-				
**bp**	0.02	-			
**is**	0.21	0.24	-		
**ls**	0.20	0.22	0.05	-	
**p**	0.18	0.15	0.08	0.04	-
