# Supplementary material for: A sophisticated case of division of labour in the trimorphic stamens of the Cassia fistula (Leguminosae) flower
Source: AoB Plants. 2021 Aug 27;13(5):plab054. doi: 10.1093/aobpla/plab054 (PMC8420109; doi:10.1093/aobpla/plab054)
Supplement: plab054_suppl_Supplementary_Dataset_S2 [file plab054_suppl_supplementary_dataset_s2.pdf]

```
####

### COMPLETE SCRIPT of Saabetal_2021 (AoBPlants)

####

### General R packages used in preliminary and final analyzes:
require(lme4)
require(nlme)
require (R2admb)
require (glmmADMB)
require(glmmTMB)
require (beeswarm)
require (MASS)
require (mgcv)
require(multcomp)
require (car)
require(ggplot2)
require(yarrr)


## Address of the folder and data files:

#setwd("XXXXXX")   #Specify the folder containing the original
article data (txt file in our case).
#getwd()
#dir()


#*******************************************************************
**************************************************
### Analyzes of the CROMATIC AND GREEN CONTRASTS across floral
parts:

## Dataset per flower:

contrast <- read.table("4b_Dataset_colour.txt",stringsAsFactors =
T,header=T)
head(contrast)
str(contrast)

plot(contrast$r.vec ~ contrast$floral.parts)

mod.r.vec <- lmer(r.vec ~ floral.parts + (1|plant),data=contrast)
summary (mod.r.vec)
anova(mod.r.vec)
summary(glht(mod.r.vec,linfct=mcp(floral.parts="Tukey")))
plot(summary(glht(mod.r.vec,linfct=mcp(floral.parts="Tukey"))),cex.a
xis=0.4)

pirateplot(r.vec ~ floral.parts,
           data=contrast,
```

```
            jitter.val=0.05,
            point.pch = 21,
            avg.line.o = 1,
            point.bg = "white",
            inf.method="ci",
            inf.p = 0.95,
            theme=0,
            pal="black",
            inf.b.o = 0.5,
            inf.f.o = 0.6,
            bean.f.o=0.05,
            bean.b.o=0,
            bean.f.col="black",
            point.o=0.3,
            ylab = "Chromatic contrast",
            xlab="Floral parts",
            ylim=c(0.1,0.4))


mod.green <- lmer(green.contrast ~ floral.parts + (1|
plant),data=contrast)
summary (mod.green)
anova(mod.green)
summary(glht(mod.green,linfct=mcp(floral.parts="Tukey")))
plot(summary(glht(mod.green,linfct=mcp(floral.parts="Tukey"))),cex.a
xis=0.4)


pirateplot(green.contrast ~ floral.parts,
            data=contrast,
            jitter.val=0.05,
            point.pch = 21,
            avg.line.o = 1,
            point.bg = "white",
            inf.method="ci",
            inf.p = 0.95,
            theme=0,
            pal="black",
            inf.b.o = 0.5,
            inf.f.o = 0.6,
            bean.f.o=0.05,
            bean.b.o=0,
            bean.f.col="black",
            point.o=0.3,
            ylab = "Green contrast",
            xlab="Floral parts",
            ylim=c(-0.2,0.42))


### Finish!


### NEXT...
```

```
#*****************************************************************
********************************************
### BEE VISITION PATTERN on flowers under different anther exclusion
treatments:

### All bee species visits:
## Dataset per flower:

cassia <-
read.table("5b_Dataset_exclusionexperiment.txt",stringsAsFactors =
T,header=T)
head(cassia)
str(cassia)


pirateplot(n.visits ~ anther.treatment,
           data=cassia,
           jitter.val=0.05,
           point.pch = 21,
           avg.line.o = 1,
           point.bg = "white",
           inf.method="ci",
           inf.p = 0.95,
           theme=0,
           pal="black",
           inf.b.o = 0.5,
           inf.f.o = 0.6,
           bean.f.o=0.05,
           bean.b.o=0,
           bean.f.col="black",
           point.o=0.3,
           ylim=c(0,10))


str(cassia)

mod.total.random1 <-  glmer.nb (n.visits~anther.treatment + (1|
locality.plant:inflorescence.code), data=cassia)
summary (mod.total.random1)

mod.total.random2 <- glmmTMB  (n.visits ~ anther.treatment + (1|
locality.plant:inflorescence.code),data=cassia,family="nbinom1")
summary (mod.total.random2)
anova(aov(mod.total.random2))

mod.total.random3 <- glmmadmb  (n.visits ~ anther.treatment + (1|
locality.plant:inflorescence.code),data=cassia,family="nbinom")
summary (mod.total.random3)
anova(aov(mod.total.random3))


### Large bee species visits (Bombus and Xylopopa species):
## Dataset per flower:
```

```
pirateplot(n.visits.largebees ~ anther.treatment,
           data=cassia,
           jitter.val=0.05,
           point.pch = 21,
           avg.line.o = 1,
           point.bg = "white",
           inf.method="ci",
           inf.p = 0.95,
           theme=0,
           pal="black",
           inf.b.o = 0.5,
           inf.f.o = 0.6,
           bean.f.o=0.05,
           bean.b.o=0,
           bean.f.col="black",
           point.o=0.3,
           ylim=c(0,10))

# Flowers without any stamens or with only the pollinating (largest)
stamen morphs had zero large bee visits.
## So, we excluded these two treatments for the second set of
analyses below:

cassia_partial <- cassia
[cassia$anther.treatment==c("a_control","b_exclusion_L","c_exclusion
_s","d_exclusion_i"),]
cassia_partial

mod.largebees.random1 <-  glmer.nb
(n.visits.largebees~anther.treatment + (1|
locality.plant:inflorescence.code), data=cassia_partial)
summary (mod.largebees.random1)
anova(mod.largebees.random1)

mod.largebees.random2 <- glmmTMB  (n.visits ~ anther.treatment + (1|
locality.plant:inflorescence.code),data=cassia_partial,family="nbino
m1")
summary (mod.largebees.random2)
anova(aov(mod.largebees.random2))

mod.largebees.random3 <- glmmadmb  (n.visits.largebees ~
anther.treatment + (1|
locality.plant:inflorescence.code),data=cassia_partial,family="nbino
m")
summary (mod.largebees.random3)
anova(aov(mod.largebees.random3))


### Finish!


### NEXT...
```

```
#***********************************************************************************************************************************
### Difference of POLLEN GRAIN GERMINATION across stamen morphs!

## Dataset per flower:

pollen_germination <- read.table("6b_Dataset_pollengermination.txt",
stringsAsFactors = T,header=T)
head(pollen_germination)
str(pollen_germination)

pirateplot(prop_sucess ~ stamen.morph,
           data=pollen_germination,
           jitter.val=0.05,
           point.pch = 21,
           avg.line.o = 1,
           point.bg = "white",
           inf.method="ci",
           inf.p = 0.95,
           theme=0,
           pal="black",
           inf.b.o = 0.5,
           inf.f.o = 0.6,
           bean.f.o=0.05,
           bean.b.o=0,
           bean.f.col="black",
           point.o=0.3,
           ylim=c(0,1))

str(pollen_germination)
y<-
cbind(pollen_germination$success.pollentube,pollen_germination$fail.
pollentube)
y

# mod.germ.1 <- glmer(y ~ stamen.morph + (1|
flower),data=pollen_germination,family="binomial")
# summary(mod.germ.1)

# mod.germ.2  <- glmmTMB  (y ~ stamen.morph + (1|
flower),data=pollen_germination,family="binomial")
# summary (mod.germ.2)

# mod.germ.3 <- glmmadmb  (y ~ stamen.morph + (1|
flower),data=pollen_germination,family="binomial")
# summary (mod.germ.3)

## Analyzes not converged because treatment 2,3 and four had zero
germinated pollen grains!



# NEXT...
```

```
#
*********************************************************************
***********************************

#######
### Differences of POLLEN SIZE across stamen morphs!

## Dataset per pollen
## Dataset per pollen size across stamen morphs:

dir()
pollen.size <- read.table("2b_Dataset_p.length.txt",stringsAsFactors
= T,header=T)
head(pollen.size)
str(pollen.size)

equ <- pollen.size[pollen.size$axes=="equatorial",]    # Equatorial
axis length of pollen grains
pol <- pollen.size[pollen.size$axes=="polar",]      # Polar axis
length of pollen grains


## Variation of equatorial axis length of pollen grains across
stamen morphs:

str(equ)

pirateplot(length._m ~ stamen.morph,
           data=equ,
           jitter.val=0.05,
           point.pch = 21,
           avg.line.o = 1,
           point.bg = "white",
           inf.method="ci",
           inf.p = 0.95,
           theme=0,
           pal="black",
           inf.b.o = 0.5,
           inf.f.o = 0.6,
           bean.f.o=0.05,
           bean.b.o=0,
           bean.f.col="black",
           point.o=0.3,
           ylim=c(20,40))


mod.equ <- lm (length._m ~ stamen.morph,data=equ)
summary (mod.equ)
anova(aov(mod.equ))
summary(glht(mod.equ,linfct=mcp(stamen.morph="Tukey")))
plot(summary(glht(mod.equ,linfct=mcp(stamen.morph="Tukey"))))
```

```
## Variation of polar axis length of pollen grains across stamen
morphs:

str(pol)

pirateplot(length._m ~ stamen.morph,
           data=pol,
           jitter.val=0.05,
           point.pch = 21,
           avg.line.o = 1,
           point.bg = "white",
           inf.method="ci",
           inf.p = 0.95,
           theme=0,
           pal="black",
           inf.b.o = 0.5,
           inf.f.o = 0.6,
           bean.f.o=0.05,
           bean.b.o=0,
           bean.f.col="black",
           point.o=0.3,
           ylim=c(20,40))


mod.pol <- lm (length._m ~ stamen.morph,data=pol)
summary (mod.pol)
anova(aov(mod.pol))

summary(glht(mod.pol,linfct=mcp(stamen.morph="Tukey")))
plot(summary(glht(mod.pol,linfct=mcp(stamen.morph="Tukey"))))



# NEXT...


#*******************************************************************
************************************

#######
### Differences of POLLEN GRAIN RESERVE across stamen morphs!

## Dataset per pollen
## Percentage of amyloplast in pollen grain sections (TEM -
transmission electronic microscopy):

dir()
pollen.TEM <-
read.table("7b_Dataset_pollenreserve.txt",stringsAsFactors =
T,header=T)
head(pollen.TEM)
str(pollen.TEM)
```

```
pirateplot(proportion.amyloplast ~ stamen.morph,
           data=pollen.TEM,
           jitter.val=0.05,
           point.pch = 21,
           avg.line.o = 1,
           point.bg = "white",
           inf.method="ci",
           inf.p = 0.95,
           theme=0,
           pal="black",
           inf.b.o = 0.5,
           inf.f.o = 0.6,
           bean.f.o=0.05,
           bean.b.o=0,
           bean.f.col="black",
           point.o=0.3,
           ylim=c(0,100))


mod.amilo <- lm (proportion.amyloplast ~
stamen.morph,data=pollen.TEM)
summary (mod.amilo)
anova(aov(mod.amilo))

#Pos-hoc
TukeyHSD(aov(mod.amilo))



###
### FINISH the data analyzes!
###
#********************************************************************
************************************************
```
